# NMR-AI: An Open Platform for NMR-Enhanced Molecular Representations and Physicochemical Property Prediction

**DOI:** 10.1021/acs.jcim.6c01026

**Published:** 2026-07-16

**Authors:** Wojciech Pietruś, Arkadiusz Leniak, Rafał Kurczab

**Affiliations:** † Department of Medicinal Chemistry, Maj Institute of Pharmacology, Polish Academy of Sciences, Smetna 12, Krakow 31-343, Poland; ‡ Department of Medicinal Chemistry, 335807 Celon Pharma S.A., Marymoncka 15, Kazun Nowy 05-152, Poland

## Abstract

Accurate prediction of physicochemical properties is increasingly limited by an information ceiling of structure-only molecular descriptors. Here, predicted ^1^H|^13^C NMR chemical shifts are transformed into fixed-length NMR vectors and concatenated with ECFP4 to form the hybrid spectral–structural representation SpectraPRINTS, enabling direct evaluation of representational complementarity across logP, logS, and logD (pH 2.6, 7.4, and 10.5), as well as the most acidic and most basic pKas. With a fixed learning protocol, SpectraPRINT reduces error for lipophilicity- and solubility-related end points (up to 39% lower RMSE vs ECFP4), while no systematic gain is observed for the most acidic and most basic macroscopic p*K*_a_ end points. The workflow is released as NMR-AI, a freely accessible web platform integrating NMR spectra prediction, descriptor construction, and property prediction, enabling interactive use and independent validation. The NMR-AI platform is accessible at https://cheminformaticsportal.if-pan.krakow.pl/.

## Graphical abstract

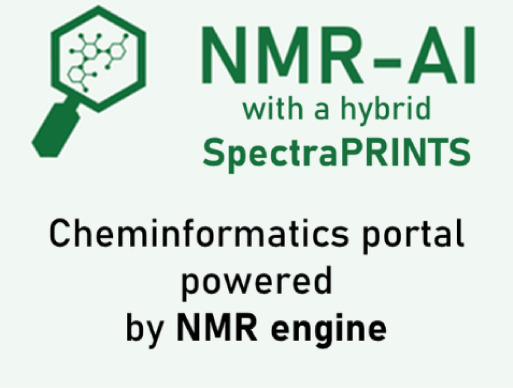

## Introduction

The accurate prediction of physicochemical properties remains a central challenge in molecular design, as key parameters such as lipophilicity, solubility, and acid–base equilibria critically determine the bioavailability and developability of small-molecule drugs.^1–5^ Over the past decades, computational approaches based on quantitative structure–property relationships (QSPR) have become indispensable, enabling rapid screening of large chemical libraries prior to synthesis and experimental characterization.^6–8^ Most contemporary QSPR models rely on molecular representations derived exclusively from chemical structure, including fragment-based descriptors, extended-connectivity fingerprints, and, more recently, graph neural networks that learn latent features directly from molecular graphs.^9,10^ However, for many physicochemical end points, gains from ever more complex structure-only representations have increasingly shown diminishing returns, consistent with an information ceiling imposed by encoding molecules purely as connectivity graphs.^11,12^ In such settings, model architecture improvements primarily repackage the same topological signal rather than add new experimentally relevant information.^13,14^ As a consequence, structure-only representations may fail to capture electronic effects, intramolecular interactions, and solution-phase phenomena that can dominate experimentally measured physicochemical properties.^15,16^

Nuclear magnetic resonance (NMR) spectroscopy provides a fundamentally different description of molecular systems that is not constrained by graph topology.^17^ NMR chemical shifts directly report on local electronic environments, reflecting electron density, hybridization state, inductive and mesomeric effects, aromatic ring currents, and hydrogen-bonding interactions.^18^ Importantly, experimentally measured and accurately predicted NMR shifts represent ensemble-averaged observables in solution, implicitly encoding conformational flexibility and solvent effects that are largely inaccessible to structure-only molecular representations.^19,20^ The NMR features used in this work were derived from predicted chemical shifts. As shown in our previous study, predicted shifts provided equal or superior performance as machine-learning inputs while removing the requirement for physical sample availability, thereby increasing applicability in early-stage molecular design.^21^ From an information-theoretic perspective, NMR-derived descriptors therefore occupy a complementary feature space, capturing electronic and environmental contributions that cannot be recovered by increasing the complexity of topological fingerprints or graph-based models.^16^ This makes NMR an attractive candidate for extending molecular representations beyond the apparent performance plateau of connectivity-based descriptors.

In a series of our recent studies, we have progressively developed the concept of using spectroscopic observables, including NMR chemical shifts and infrared vibrational frequencies, as molecular descriptors for machine learning.^21–25^ Infrared spectra were shown to provide rich, transferable representations of molecular structure and physicochemical properties,^23,25^ while binned NMR spectra could be transformed into fixed-length numerical vectors suitable for quantitative structure–property modeling.^21,22,24^ In our first NMR-based QSPR study,^21^ experimental ^1^H NMR spectra were converted into fixed-length numerical vectors and used to predict chromatographically derived logD values for a smaller proprietary data set measured at pH 2.6, 7.4, and 10.5. The best model reached RMSE 0.66 for CHI logD at pH 7.4, but this result depended on experimental spectra, extensive preprocessing, dimensionality reduction, and computationally intensive validation. In the next step,^22^ this experimental bottleneck was addressed by replacing measured spectra with computer-generated ^1^H NMR inputs obtained using DFT, JEOL JASON, and NMRshiftDB2. Predicted spectra generated with NMRshiftDB2 and JEOL JASON gave the best downstream logD performance, with RMSE values as low as 0.76, whereas DFT-derived spectra were less effective and substantially more expensive to generate. Most recently,^24^ predicted ^1^H and ^13^C NMR vectors were evaluated as standalone and fused spectral representations for logD modeling and compared with ECFP4. The best fused 1H|13C model achieved RMSE 0.57 and *Q* ^2^ 0.76, approaching the ECFP4 benchmark while using a substantially lower-dimensional input. However, these earlier studies primarily compared NMR-based and structure-based representations as alternative descriptors. The present work extends this line of research by directly testing representational complementarity: if NMR-derived vectors were redundant with ECFP4, their simple concatenation with ECFP4 would not be expected to improve predictive performance systematically. Therefore, the SpectraPRINTS representation was designed as a direct spectral–structural concatenation and evaluated across logP, logS, logD, and p*K*_a_ end points.

In brief, SpectraPRINTS combine binned one-dimensional ^1^H and ^13^C NMR vectors with ECFP4 in a single fixed-length spectral-structural representation (Figure 1), yielding descriptor sizes compatible with standard machine-learning workflows. Descriptor construction and dimensionality are described in the (Supporting Information Sections 3.2–3.4). Despite these methodological advances, the practical adoption of machine learning-based molecular models remains limited. In many cases, predictive models and descriptors are released primarily as standalone code repositories, requiring substantial expertise in cheminformatics and machine learning to deploy, adapt, and validate.^26–29^ This creates a significant accessibility gap between methodological development and routine use, particularly for experimental and medicinal chemists without extensive programming backgrounds.^28,30^ At the same time, commercially available cheminformatics platforms typically provide only partial coverage of such workflows, with limited support for various representations and machine-learning-driven property prediction. Building on this foundation, the present work shifts focus from representation development to representation complementarity, testing whether NMR-derived features can systematically augment established structure-based fingerprints and overcome the apparent performance plateau of connectivity-only molecular representations. To lower the deployment barriers, the resulting workflow is provided through a freely accessible platform.

**Figure 1:**
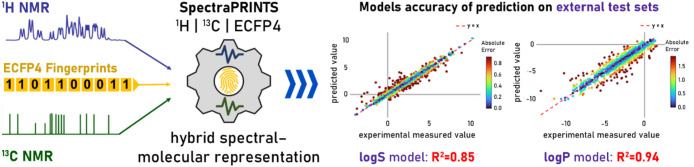
Construction scheme of the SpectraPRINTS molecular representation and its application to physicochemical property prediction. Predicted one-dimensional ^1^H and ^13^C NMR spectra are transformed into fixed-length spectral descriptors and combined with ECFP4 fingerprints to form a hybrid spectral–structural representation suitable for machine learning. The right-hand panels show representative prediction results for logP and logS models, comparing predicted values against experimental reference data for compounds in an independent held-out test set not used during model training or hyperparameter optimization, thereby reflecting true out-of-sample predictive performance.

## Materials and Methods

### Data Sources, Curation, and End Point Definitions

All data sets were curated using an in-house RDKit-based Python workflow. Only single-component organic molecules were retained. Multicomponent records, including salts, mixtures, and solvates, were removed, as were compounds containing inorganic elements or metal ions. This filtering was required because the NMR-derived descriptors used in this work were based on predicted one-dimensional ^1^H and ^13^C spectra and were, therefore, not applicable to inorganic or salt-containing entries. A strict salt-removal policy was adopted to reduce experimental variability unrelated to the intrinsic molecular structure, particularly for solubility and pH-dependent partitioning. The curated data sets comprised 13962 compounds for logP, 8300 for intrinsic aqueous solubility (logS), 1610 for logD at pH 2.6, 5694 for logD at pH 7.4, 1514 for logD at pH 10.5, 3073 for the most acidic p*K*_a_, and 3550 for the most basic p*K*_a_ (Table 1). LogP was treated as the neutral octanol/water partition coefficient, logS as intrinsic aqueous solubility, and logD as the pH-dependent octanol/water distribution coefficient measured at three pH conditions. Acid–base behavior was modeled using macroscopic labels corresponding to the most acidic and most basic p*K*_a_ values for each molecule. Because these observables collapse multiple protonation microstates and tautomeric forms into a single value, they provide a less direct target for global molecular representations than the remaining physicochemical end points. Additional data set-specific curation details, end point definitions, and distribution analyses are provided in the (Supporting Information Sections 2.1–2.3).

**Table 1:** Overview of Curated Datasets and Physicochemical End Points Used for Model Development

End point	Data source	No. of molecules
logP	Opera data set^31^	13962
logS	Opera data set^31^	8300
logD (pH 2.6)	Celon Pharma^21^	1610
logD (pH 7.4)	MoleculeNet^32^ + Celon Pharma^21^	5694
logD (pH 10.5)	Celon Pharma^21^	1514
most acidic p*K*_a_	Opera data set^31^	3073
most basic p*K*_a_	Opera data set^31^	3550

### Molecular Representations

Molecular structures were encoded using three alternative input representations. The first consisted of ECFP4 fingerprints^9^ generated with RDKit^33^ as 2048-bit Morgan fingerprints with radius 2 and chirality enabled. The second consisted of NMR-only vectors derived from predicted one-dimensional ^1^H and ^13^C chemical shifts. Chemical shifts were predicted using a HOSE-code-based^34^ workflow derived from nmrshiftdb2 ^20^ and transformed into fixed-length numerical vectors by histogram-like binning. The ^1^H region from −1 to 16 ppm and the ^13^C region from −10 to 230 ppm were each divided into 200 equal-width bins, yielding a 400-dimensional NMR representation. The third representation consisted of SpectraPRINTS, defined here as the direct concatenation of the ^1^H and ^13^C NMR vectors with ECFP4, resulting in a 2448-dimensional spectral-structural descriptor. This setup allowed ECFP4, NMR-only, and SpectraPRINTS representations to be benchmarked under an identical learning protocol. The NMR descriptor generation workflow was adapted from our previous study,^24^ with the same HOSE-code-based shift prediction framework and fixed-bin spectral encoding applied here to a broader benchmark of physicochemical end points; implementation details for descriptor construction are provided in the (Supporting Information Sections 3.1–3.5).

### Model Training and Evaluation

All predictive models were implemented as one-dimensional convolutional neural networks for regression. To ensure that performance differences reflected the information content of the molecular representations rather than model flexibility, the same modeling framework was applied across all experiments. Model architecture and training hyperparameters were optimized jointly using Optuna,^35^ including the number of convolutional and fully connected layers, filter counts, kernel sizes, dropout rates, activation functions, optimizer settings, and learning-rate scheduling. Training was performed with a supervised mean-squared-error objective, with early stopping applied to the validation loss to limit overfitting and Optuna pruning used to terminate underperforming trials. The full data set was partitioned once into a training set comprising 90% of samples and an independent held-out test set comprising the remaining 10%, and all hyperparameter optimization procedures were restricted to the training portion of the data. Within the training set, 10-fold cross-validation was used during optimization, whereas an internal validation split was used to control early stopping and learning-rate scheduling during final model fitting. After model selection, the final model was retrained on the full training set and evaluated once on the untouched test set. Predictive performance was assessed using RMSE as the primary error metric, *Q*² as a cross-validated estimate of predictive robustness, and *R* ^2^ as a descriptive measure of goodness of fit on the final model. Applicability Domain was evaluated at two complementary levels. First, the end point-specific chemical space was characterized using molecular weight, topological polar surface area, heavy atom count, and number of rotatable bonds to define the empirical descriptor ranges represented by the training data (Supporting Information Section 2.5). Second, model-level reliability was assessed using an embedding-based Applicability Domain analysis, in which Mahalanobis distances were calculated in the latent representation of the trained neural network and interpreted together with standardized residuals (Supporting Information Section 6.2). The complete chemical-space characterization and end point-specific Applicability Domain plots are provided in the Supporting Information. Detailed optimization settings, implementation parameters, and extended evaluation procedures are provided in the (Supporting Information Sections 4–5 and 9.1.5). To quantify sensitivity to data partitioning, fold-wise variability was reported for the 10-fold cross-validation procedure. For each end point and molecular representation, mean RMSE and *Q* ^2^ values were accompanied by standard deviations and fold-to-fold ranges. This analysis was used to assess whether the observed ranking of representations was stable across validation folds rather than being driven by a single favorable partition (Supporting Information, Section 6.2).

## Results and Discussion

To directly test whether NMR-derived descriptors provide information complementary to structure-only fingerprints, we designed a representation-focused benchmarking strategy spanning a diverse set of physicochemical end points. The analysis encompasses multiple independent properties governing lipophilicity, solubility, and acid–base behavior, including logP, logS, logD (evaluated at pH 2.6, 7.4, and 10.5), and the most acidic and basic p*K*_a_, thereby probing the generality of the proposed representation across chemically and environmentally distinct regimes. These end points were selected to cover properties dominated by different underlying factors, ranging from largely hydrophobic interactions to protonation-dependent equilibria. For each property, three molecular representations were evaluated: conventional extended-connectivity fingerprints (ECFP4), NMR-only descriptors constructed from binned ^1^H and ^13^C chemical shifts, and SpectraPRINTS defined as a hybrid representation formed by their direct concatenation with ECFP4. ECFP4 was selected as a mature and widely adopted topological representation that has repeatedly shown strong baseline performance in physicochemical property prediction, making it a suitable reference for assessing representational complementarity.^9–37^ To ensure that observed performance differences reflect representational content rather than model flexibility, the machine-learning architecture, training protocol, and evaluation metrics were intentionally kept fixed across all experiments (detailed description in the Supporting Information Section 4). Predictive performance was quantified using root-mean-square error (RMSE) to retain direct interpretability in physicochemical units (Supporting Information Section 5). Under this design, representational redundancy constitutes a falsifiable hypothesis: if NMR-derived descriptors merely re-encode information already present in structure-based fingerprints, their combination would not be expected to yield systematic improvements across independent properties and protonation conditions.

Across lipophilicity- and solubility-related end points, the hybrid representation improved predictive performance relative to the structure-only baseline on the independent held-out test set, whereas no systematic gain was observed for the most acidic and most basic macroscopic p*K*_a_ end points (Table 2 and Table S1). These results demonstrate that spectral and structural descriptors encode complementary, nonredundant information that translates into superior generalization performance (Figure 1). Full benchmarking results are reported in the (Supporting Information Table S1). Model performance and reliability were visualized using paired diagnostic panels comprising a parity plot and an embedding-based applicability domain (eAD) analysis in Figures S12–S18. These analyses, together with the chemical-space characterization of each end point-specific data set, are reported in the (Supporting Information Section 6.3). For each end point and model variant, these panels provide an interpretable summary of predictive accuracy on the independent test set together with a complementary assessment of whether predictions are produced within the model’s learned domain of applicability.

**Table 2:** Predictive Performance of Machine-Learning Models Trained on Three Molecular Representations: ECFP4 Fingerprints, NMR-Derived Spectral Descriptors Constructed from Binned ^1^H and ^13^C Chemical Shifts (^1^H|^13^C), and SpectraPRINTS, Defined as Their Concatenation with ECFP4 (^1^H|^13^C|ECFP4), Evaluated across logP, logD (pH 2.6, 7.4, and 10.5), and logS^b^

	RMSE			
Property	ECFP4	1H|13C	SpectraPRINTS	**% of enhancement** ^a^
**logP**	0.68	0.66	0.47	31
**logD (2.6)**	0.76	0.52	0.51	33
**logD (7.4)**	0.74	0.85	0.62	16
**logD (10.5)**	0.76	0.64	0.46	39
**logS**	1.15	1.07	0.93	19

a Percentage enhancement is calculated as the relative RMSE reduction of the hybrid representation compared with ECFP4: % enhancement = 100­(RMSE_ECFP4 - RMSE_1H|13C|ECFP4)/RMSE_ECFP4.

b Model performance is primarily assessed using RMSE calculated on an independent held-out test set (10% of the data), with cross-validated metrics reported for reference.

For neutral lipophilicity (logP), the hybrid model reduced the test-set RMSE from 0.68 for ECFP4 alone to 0.47, corresponding to a 31% reduction in prediction error while simultaneously increasing the coefficient of determination to *R* ^2^ = 0.94. Comparable improvements were observed for the pH-dependent lipophilicity. For logD at pH 2.6, the hybrid representation achieved a substantial reduction in the test set RMSE from 0.76 to 0.51 (33% improvement), accompanied by an increase in *R* ^2^ from 0.78 to 0.80. For logD at pH 7.4, the test set RMSE decreased from 0.74 to 0.62 (16% improvement), with a corresponding increase in *R* ^2^ from 0.62 to 0.67.

Notably, for logD at pH 10.5, the hybrid model yielded the most pronounced gain in generalization performance, reducing the test set RMSE from 0.76 to 0.46 (39% improvement) and increasing *R* ^2^ from 0.58 to 0.83, despite only marginal differences observed during cross validation. This result highlights the importance of independent test set evaluation for reliably assessing model performance.

For intrinsic solubility (log S), the hybrid representation reduced the test set RMSE from 1.15 to 0.93 (19% improvement), with a concurrent increase in *R* ^2^ from 0.78 to 0.86, confirming the added value of combining spectral and structural information. In contrast, for acid and base p*K*_a_ prediction, no consistent improvement was observed in the held-out test set. In these cases, the hybrid representation yielded test set errors comparable to or slightly higher than those obtained with structure-based descriptors alone, suggesting that NMR-derived features provide limited additional information for p*K*_a_ prediction within the investigated data sets.

For the most acidic and most basic macroscopic p*K*_a_ end points, the weaker contribution of NMR-derived information is consistent with the nature of the target labels themselves. These observables collapse multiple protonation microstates, tautomeric forms, and site-specific equilibria into a single scalar value, thereby weakening the direct correspondence between local NMR observables and the reported end point. As a result, although the hybrid representation can yield accurate predictions for selected chemotypes, performance gains remain less systematic than for lipophilicity- and solubility-related properties. Importantly, the NMR-only representation remained competitive but generally inferior to the full spectral-structural model, indicating that NMR-derived features contribute complementary rather than redundant information. Chemical-space summaries and applicability-domain analyses are provided in the (Supporting Information Section 2.5 and Figures S5–S11).

Taken together, these results highlight that the predictive advantage of the hybrid NMR-structural representation arises from its ability to encode physicochemical information that is orthogonal to the molecular topology. The largest and most consistent improvements are observed for properties governed by distributed electronic effects and solution-phase behavior, such as lipophilicity and solubility, where NMR chemical shifts naturally reflect the underlying electronic environment. In contrast, acid–base equilibria expose intrinsic limitations of simplified labeling schemes: apparent p*K*_a_ values collapse multiple microstates, protonation sites, and tautomeric forms into a single observable, obscuring the direct relationship between the molecular representation and experimental measurement. Addressing such end points in a fully general manner is therefore likely to require microstate-aware modeling strategies that explicitly account for speciation and population-weighted observables, rather than further refinement of global molecular descriptors alone. Importantly, these limitations do not undermine the central conclusion of this work but instead delineate the regime in which NMR-derived descriptors provide the maximal benefit. By extending molecular representations beyond connectivity-defined graphs, NMR-based features expand the effective information content available to machine-learning models and enable systematic improvements for a broad class of physicochemical properties that have approached a performance plateau under structure-only representations.

In addition to benchmarking molecular representations, this work addresses a persistent gap between methodological advances in molecular machine learning and their practical adoption by providing an openly accessible platform that integrates all major stages of NMR-enhanced molecular modeling (Figure 2). Unlike existing tools that address isolated stages of molecular modeling, NMR-AI is an open web platform that integrates structure handling, NMR spectra prediction, descriptor generation, machine learning input construction, and physicochemical property prediction within an integrated environment. Implementation details and the end-to-end workflow are provided in the (Supporting Information Sections 8–9). Predicted ^1^H and ^13^C spectra are generated using fragment-based HOSE-code methodology calibrated against reference data from nmrshiftdb2 ^20^ and can be directly transformed into binned NMR descriptors (SpectraPRINTS) or visualized at atomic resolution. This tight coupling among spectral prediction, representation construction, and property inference enables users to move seamlessly from molecular structure to model-ready features and predicted properties without external preprocessing or proprietary software.

**Figure 2:**
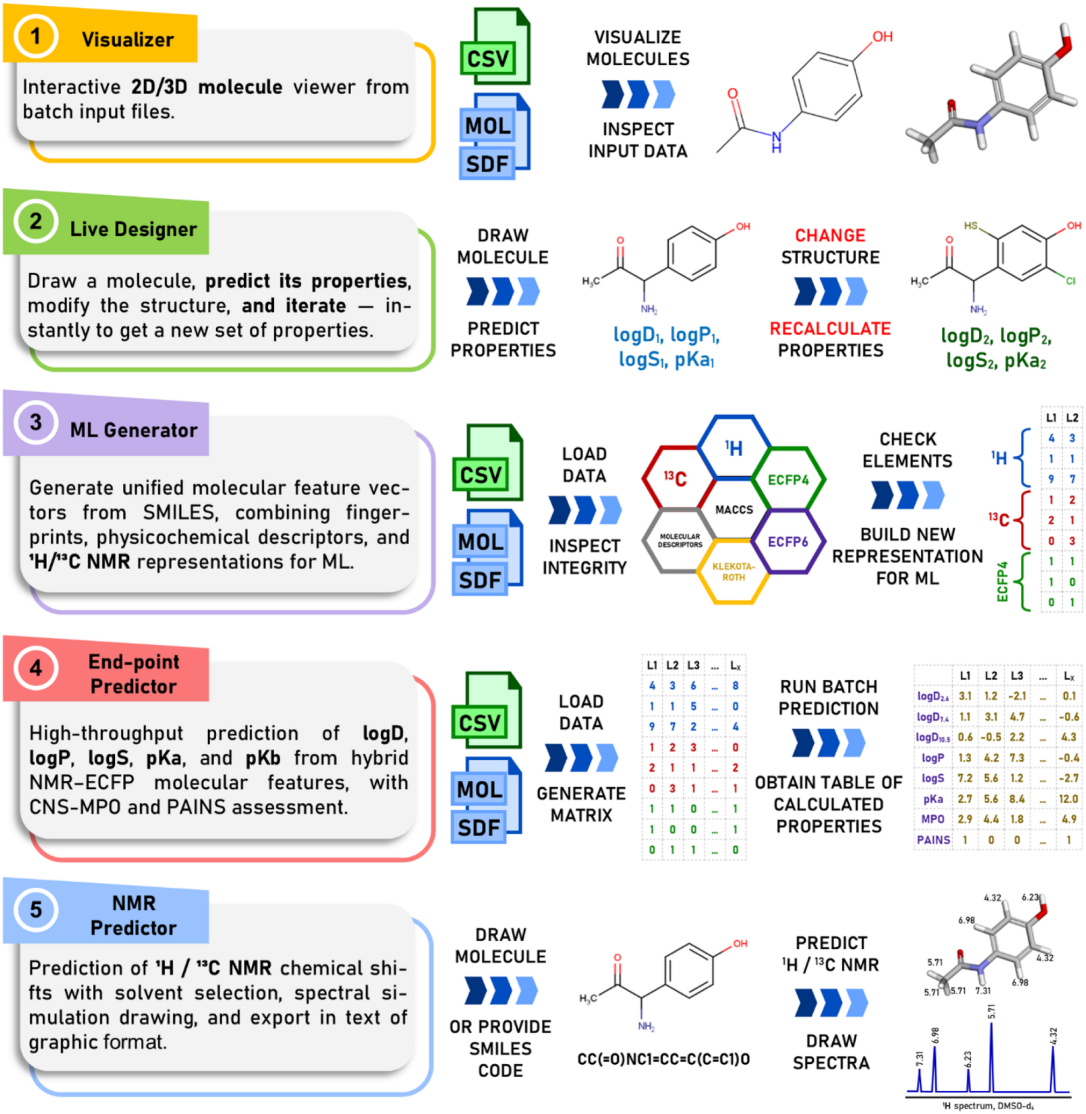
Conceptual overview of the NMR-AI platform, illustrating the modular yet integrated workflow for NMR-enhanced molecular modeling. The platform combines molecular visualization, NMR spectra prediction, construction of hybrid spectral–structural representations, and machine-learning-based prediction of physicochemical properties within a single environment. Individual modules operate on a common molecular input and are designed to support both interactive molecular design and high-throughput batch prediction, enabling seamless transition from molecular structure to model-ready representations and predicted properties.

Importantly, NMR-AI provides functionality that is typically distributed across multiple commercial cheminformatics packages, while remaining freely available and openly accessible. The platform supports a broad range of molecular representations, including circular fingerprints, topological and atom-pair fingerprints, Klekota–Roth fingerprints, and physicochemical descriptor blocks, all of which can be flexibly combined with NMR-derived SpectraPRINTS for custom machine-learning workflows. A live molecular design environment enables real-time prediction of physicochemical properties for successive structural modifications, allowing interactive exploration of structure–property relationships during compound optimization. Such real-time, representation-aware molecular design capabilities are not commonly available in existing open-access platforms and are typically restricted to closed commercial ecosystems. In addition to direct property prediction, the platform provides derived decision metrics commonly used in medicinal chemistry, including PAINS substructure alerts^38^ and CNS-MPO^39^ scores computed from predicted physicochemical properties and standard structural descriptors. By lowering the barrier to advanced NMR-enhanced molecular modeling, NMR-AI enables broader adoption of hybrid spectral–structural representations in both academic and applied research settings.

## Conclusions

In summary, the SpectraPRINTS introduced herein, defined as a hybrid ^1^H|^13^C|ECFP4 representation, provide information complementary to conventional structure-based fingerprints and reduce prediction error for key lipophilicity- and solubility-related end points relative to structure-based descriptors alone. In contrast, p*K*_a_ shows no systematic gain under macrostate labels that collapse multiple microstates and tautomers, indicating that further progress for acid–base equilibria will require microstate-aware modeling rather than refinement of global descriptors alone. Beyond benchmarking, NMR-AI provides a freely accessible integrated web platform that couples spectra prediction, descriptor generation, ML-ready feature export, and property prediction, and includes a live molecular design environment enabling real-time evaluation of physicochemical properties across successive structural modifications, thereby lowering the practical barrier to adoption and independent validation. By combining complementary molecular representations with immediate, interactive access to predictive models, NMR-AI bridges the gap between methodological advances in molecular machine learning and their routine use in molecular design.

## Supplementary Material



## Data Availability

The source code for the machine-learning training and optimization workflow used in this study is openly available at https://github.com/Prospero1988/NMR-AI_part4. The NMR-AI platform described in this work is accessible at https://cheminformaticsportal.if-pan.krakow.pl/. Registration is required for full access to prediction modules and interactive functionality.

## Notes

Detailed description of data sets and data curation procedures; definition of physicochemical end points; construction of SpectraPRINTS descriptors; concatenated spectral–structural representations; machine learning models and training protocols; cross-validation and evaluation metrics; extended benchmarking results; applicability domain analysis; software implementation details and library versions; additional figures and tables. Additional references cited only in the Supporting Information are listed therein (PDF)

## Abbreviations

^1^H NMR: proton nuclear magnetic resonance
^13^C NMR: carbon-13 nuclear magnetic resonance
CNS-MPO: central nervous system multiparameter optimization
ECFP4: extended-connectivity fingerprint with radius 2
HOSE: hierarchical organization of spherical environments
IR: infrared
ML: machine learning
MW: molecular weight
NMR: nuclear magnetic resonance
PAINS: pan-assay interference compounds
QSPR: quantitative structure–property relationships
RMSE: root-mean-square error
TPSA: topological polar surface area
