# Supplementary material for: NMR-AI: An Open Platform for NMR-Enhanced Molecular Representations and Physicochemical Property Prediction
Source: J Chem Inf Model. 2026 Jul 16;66(14):8318–24. doi: 10.1021/acs.jcim.6c01026 (PMC13417872; doi:10.1021/acs.jcim.6c01026)
Supplement: Supplementary file 1 [file ci6c01026_si_001.pdf]

NMR-AI: An Open Platform for NMR-Enhanced Molecular Representations and Physicochemical Property Prediction

*Wojciech Pietruś,[a] Arkadiusz Leniak,[b] and Rafał Kurczab[a]**

[a] Department of Medicinal Chemistry, Maj Institute of Pharmacology, Polish Academy of Sciences, Smetna 12, 31-343 Krakow, Poland

[b] Department of Medicinal Chemistry, Celon Pharma S.A., Marymoncka 15, 05-152 Kazun Nowy, Poland

Content

Content 1
1 Overview of the Supporting Information 4
2 Datasets and Physicochemical Endpoints 5
2.1 Data sources and curation 5
2.1.1 Lipophilicity (logP) 5
2.1.2 Solubility (logS) 6
2.1.3 pH-dependent distribution coefficients (logD) 6
2.1.4 Acid-base equilibria: most acidic and most basic pKa 7
2.2 Definition of physicochemical endpoints 7
2.2.1 Neutral lipophilicity (logP) 7
2.2.2 Intrinsic aqueous solubility (logS) 7
2.2.3 pH-dependent distribution coefficient (logD) 7
2.2.4 Acid–base equilibria: most acidic and most basic pKa 8
2.3 Scope and limitations of endpoint definitions 8

2.4 Statystical analysis of datasets 8
2.5 Definition of the applicability domain 12
2.5.1 Overall dataset composition 12
2.5.2 Distribution of physicochemical descriptors 12
2.5.3 Definition of the applicability domain 17
3 Molecular Representations 17
3.1 ECFP4 fingerprint generation 17
3.2 NMR vectors generation 18
3.3 Construction of SpectraPRINTS descriptors 18
3.4 SpectraPRINTS dimensionality and normalization 19
3.5 Concatenated spectral–structural representations 19
4 Machine Learning Models and Training Protocol 19
4.1 Model architectures 19
4.2 Training procedure and hyperparameters 20
4.3 Cross-validation and data splitting strategy 20
5 Model Evaluation Metrics 21
5.1 Root-mean-square error (RMSE) 21
5.2 Cross-validated coefficient of determination (Q^2) 22
5.3 Coefficient of determination (R^2) 22
6 Extended Benchmarking Results 22
6.1 Full performance tables for all endpoints 22
6.2 Parity plots and error distributions 24
6.3 Endpoint-specific performance analysis 28
7 Acid–Base Modeling and pKa/pKb Handling 30
7.1 Acid–base site classification 30
7.2 Selection of most acidic and most basic pKa values 30
7.3 Limitations of macroscopic pKa labeling 31
8 NMR-AI Platform Description 32
8.1 Molecular input and representation generation 32
8.2 NMR spectra prediction and SpectraPRINTS construction 32
8.3 Property prediction and derived decision metrics 33
8.4 Interactive live molecular design 34
8.5 Visualization and data export 34

9 Software Implementation and Reproducibility 35
9.1 Software libraries and versions 35
9.1.1 Web application framework 35
9.1.2 Cheminformatics and molecular representations 35
9.1.3 NMR spectra prediction 36
9.1.4 Acid–base classification 36
9.1.5 Machine learning 36
9.2 Reproducibility 37
10 Supplementary References 38

1 Overview of the Supporting Information

This Supporting Information provides a detailed description of the datasets, molecular representations, physicochemical endpoints, analysis procedures underlying the NMR-AI platform, and the predictive models reported in the main manuscript. The aim of this document is to ensure methodological transparency, reproducibility, and clear delineation of the chemical space and applicability domain associated with the presented results.

Section 2 describes the data sources, curation procedures, and definitions of the physicochemical endpoints considered in this study, including neutral lipophilicity (logP), intrinsic aqueous solubility (logS), pH-dependent distribution coefficients (logD), and macroscopic acid–base dissociation constants. Statistical analyses and value distributions are provided to characterize the range and balance of each dataset. **Section 3** details the molecular representations employed, including structure-based fingerprints, physicochemical descriptors, and NMR-derived SpectraPRINTS features, as well as their dimensionality and normalization. The construction of concatenated spectral–structural representations is described to support systematic comparison of individual and combined feature spaces. **Section 4** summarizes the machine learning models and training protocols, including model architectures, data splitting strategies, and validation procedures. **Section 5** defines the evaluation metrics used to assess model performance, while **Section 6** provides extended benchmarking results, including full performance tables, parity plots, and endpoint-specific analyses. **Section 7** focuses on acid–base modeling, including ionizable site classification, selection of most acidic and most basic macroscopic pKa values, and limitations arising from microstate collapsing and tautomeric ambiguity. **Section 8** presents a detailed description of the NMR-AI platform, outlining its modular design, data processing workflow, prediction capabilities, interactive molecular design features, and visualization tools. Finally, **Section 9** documents the software implementation, libraries, and versions used, together with reproducibility considerations.

Together, this Supporting Information complements the main manuscript by providing the methodological details required for independent assessment, reproduction, and extension of the presented work.

2 Datasets and Physicochemical Endpoints

2.1 Data sources and curation

All datasets were curated using a in-house Python workflow (RDKit-based) designed to ensure chemical consistency and compatibility with NMR-based molecular representations. Only single-component organic molecules were retained for subsequent analysis. Multicomponent records, including salts, mixtures, and solvates, were excluded, and compounds containing inorganic elements or metal ions were removed.

Restriction to organic molecules was required because the NMR-derived representations employed in this work are based on predicted one-dimensional ^{1}H and ^{13}C spectra. Inorganic compounds and purely inorganic fragments do not produce meaningful ^{1}H or ^{13}C NMR signals and therefore cannot be represented within the SpectraPRINTS framework. Retaining such entries would lead to empty spectral descriptors and was consequently avoided.

A strict salt-removal policy was adopted rather than desalted fragment retention. Although counterions do not alter the underlying molecular topology, different salt forms of the same parent compound may exhibit substantially different experimentally measured physicochemical properties, particularly solubility and pH-dependent distribution coefficients. Retaining salt forms would therefore introduce additional variability unrelated to intrinsic molecular structure or electronic environment, increasing noise in supervised learning tasks. To minimize such confounding effects, all salt-containing records were removed and only neutral or intrinsically charged organic molecules without counterions were considered.

This curation strategy yields a chemically homogeneous dataset aligned with both the requirements of NMR-based representations and the objective of benchmarking intrinsic physicochemical property prediction. This filtering strategy intentionally removes information related to pharmaceutical salt forms and formulation-dependent effects. While such effects are relevant at later stages of drug development, they are outside the scope of intrinsic, structure-driven property modeling addressed in this work.

2.1.1 Lipophilicity (logP)

The logP model was trained using a curated subset of the OPERA dataset for physicochemical property prediction.[1] Experimental logP values were collected from diverse literature sources and previously harmonized within the OPERA framework. The dataset comprises 13962

organic molecules after curation and filtering. Only neutral organic compounds were retained to ensure consistency with the definition of intrinsic lipophilicity. Molecular structures were standardized and represented using concatenated ECFP4 fingerprints and NMR-derived SpectraPRINTS descriptors.

2.1.2 Solubility (logS)

Intrinsic aqueous solubility (logS) models were developed using the OPERA solubility dataset,[1] which aggregates experimentally measured solubility values from multiple public sources. The final curated dataset contains 8300 organic molecules. To reduce experimental heterogeneity, salts and multi-component systems were excluded, and only single-component organic molecules were retained. The same molecular representations and data splitting strategy as for logP were applied. Solubility values are reported as logarithmic molar solubility under intrinsic conditions.

2.1.3 pH-dependent distribution coefficients (logD)

pH-dependent distribution coefficients (logD) were modeled using multiple datasets corresponding to different protonation regimes. Three independent datasets were used for pH-dependent lipophilicity, corresponding to logD at pH 2.6, 7.4, and 10.5.

- **logD pH 2.6:** Dataset comprising 1610 molecules, sourced from an internal Celon Pharma drug design database.[2]
- **logD pH 7.4:** Combined dataset consisting of 5694 molecules, including 4200 compounds from the MoleculeNet benchmark and 1494 additional compounds from the Celon Pharma dataset.[2,3]
- **logD pH 10.5:** Dataset comprising 1514 molecules, sourced exclusively from the Celon Pharma internal database.[2]

All datasets were filtered to retain only organic, single-component molecules, as inorganic compounds and salts do not produce meaningful ^{1}H or ^{13}C NMR spectra and may introduce variability due to counterion effects. The same representation scheme and data splitting protocol were used across all logD models.

2.1.4 Acid-base equilibria: most acidic and most basic pKa

Acid–base behavior was modeled using two distinct datasets derived from the same literature source,[1] but treated as separate endpoints:

- **Most acidic pKa:** Dataset comprising 3073 molecules, where each molecule is annotated with the experimentally determined pKa of its most acidic ionizable site.
- **Most basic pKa:** Dataset comprising 3550 molecules, where each molecule is annotated with the pKa of its most basic ionizable site.

Although both datasets originate from the same underlying source, they were handled independently due to their different physicochemical interpretation and label distributions. Importantly, these macroscopic pKa labels collapse multiple microstates, protonation sites, and tautomeric forms into a single observable value. This limitation is discussed in detail in the main text and motivates the observed lack of systematic performance gains for pKa prediction using global molecular representations.

2.2 Definition of physicochemical endpoints

2.2.1 Neutral lipophilicity (logP)

The neutral partition coefficient (logP) is defined as the logarithm of the equilibrium concentration ratio of a neutral compound between n-octanol and water. LogP reflects intrinsic hydrophobicity and is independent of ionization state. In this work, logP values correspond to experimentally measured neutral species and were used as a reference endpoint dominated primarily by hydrophobic and dispersive interactions.

2.2.2 Intrinsic aqueous solubility (logS)

Intrinsic solubility (logS) is defined as the logarithm of the molar solubility of a compound in water under conditions where ionization effects are minimized. LogS captures contributions from crystal lattice energy, molecular polarity, and solvation, and is treated here as an intrinsic property of the neutral parent compound.

2.2.3 pH-dependent distribution coefficient (logD)

The distribution coefficient (logD) is defined as the logarithm of the concentration ratio of all molecular species (ionized and neutral) between octanol and water at a specified pH. Unlike

logP, logD explicitly depends on acid–base equilibria and reflects the combined effects of lipophilicity and ionization.

In this study, logD values were modeled at three pH conditions:

- **pH 2.6** (acidic),
- **pH 7.4** (physiological),
- **pH 10.5** (basic),

thereby probing ionization-dependent lipophilicity across chemically and biologically relevant environments.

2.2.4 Acid–base equilibria: most acidic and most basic pKa

Acid–base behavior was characterized using macroscopic pKa values. For each molecule, two distinct endpoints were defined:

- **Most acidic pKa**, corresponding to the lowest pKa value among all ionizable sites.
- **Most basic pKa**, corresponding to the highest pKa value among all basic ionizable sites.

These macroscopic pKa values represent experimentally accessible observables but inherently collapse multiple protonation microstates, tautomeric forms, and site-specific equilibria into a single scalar value. As a result, they do not provide explicit information on microstate populations or site-specific protonation behavior.

2.3 Scope and limitations of endpoint definitions

All physicochemical endpoints used in this study correspond to experimentally measured macroscopic quantities. While these values are directly relevant for molecular design and compound prioritization, they do not explicitly resolve microstate-level phenomena such as site-specific protonation, tautomerism, or conformational equilibria. These limitations are particularly relevant for acid–base properties and are discussed in the context of model performance and representation complementarity.

2.4 Statystical analysis of datasets

The distributions of all physicochemical endpoints used in this study were analyzed to assess their numerical ranges, central tendencies, and degree of heterogeneity (Figures S1–S4). Across

all datasets, the observed value distributions are continuous, non-uniform, and in several cases exhibit pronounced skewness, reflecting the chemically diverse nature of the underlying compound collections.

Neutral lipophilicity values (logP) span a broad range, extending from strongly hydrophilic to highly lipophilic compounds (approximately −5 to >10) (Figure S1). The distribution is unimodal with a long right tail and a median 2.0, consistent with datasets enriched in drug-like molecules but retaining significant chemical diversity. The heavy-tailed shape indicates the presence of highly lipophilic outliers, which are known to challenge predictive modeling and justify the use of robust error metrics.

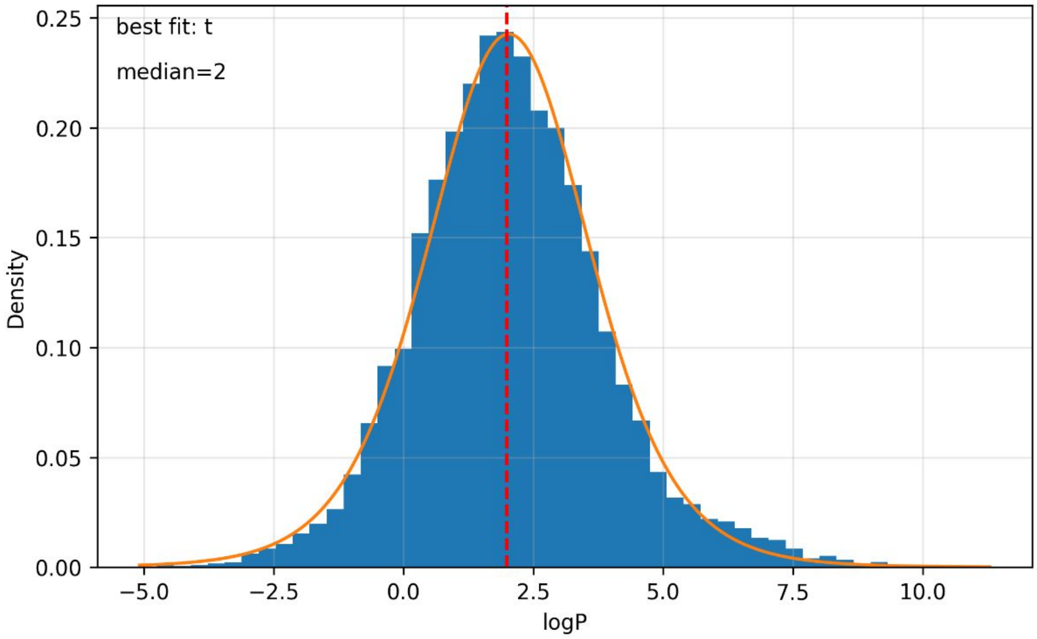

Figure S1. Distribution of logP values with fitted probability density and median.

pH-dependent distribution coefficients (logD) display distinct distributional characteristics depending on the protonation regime (Figure S2). At physiological pH (logD pH 7.4) (Figure S2B), values are centered around a median of 2.4 and exhibit moderate right skewness, reflecting the combined influence of lipophilicity and ionization. At acidic conditions (logD pH 2.6) (Figure S2A), the distribution shifts toward lower values (median = 0.85), consistent with increased protonation of basic functional groups. In contrast, at basic conditions (logD pH 10.5) (Figure S2C), the distribution shifts toward higher values (median = 2.8) and shows heavier tails, indicating substantial variability in deprotonation behavior across chemical classes.

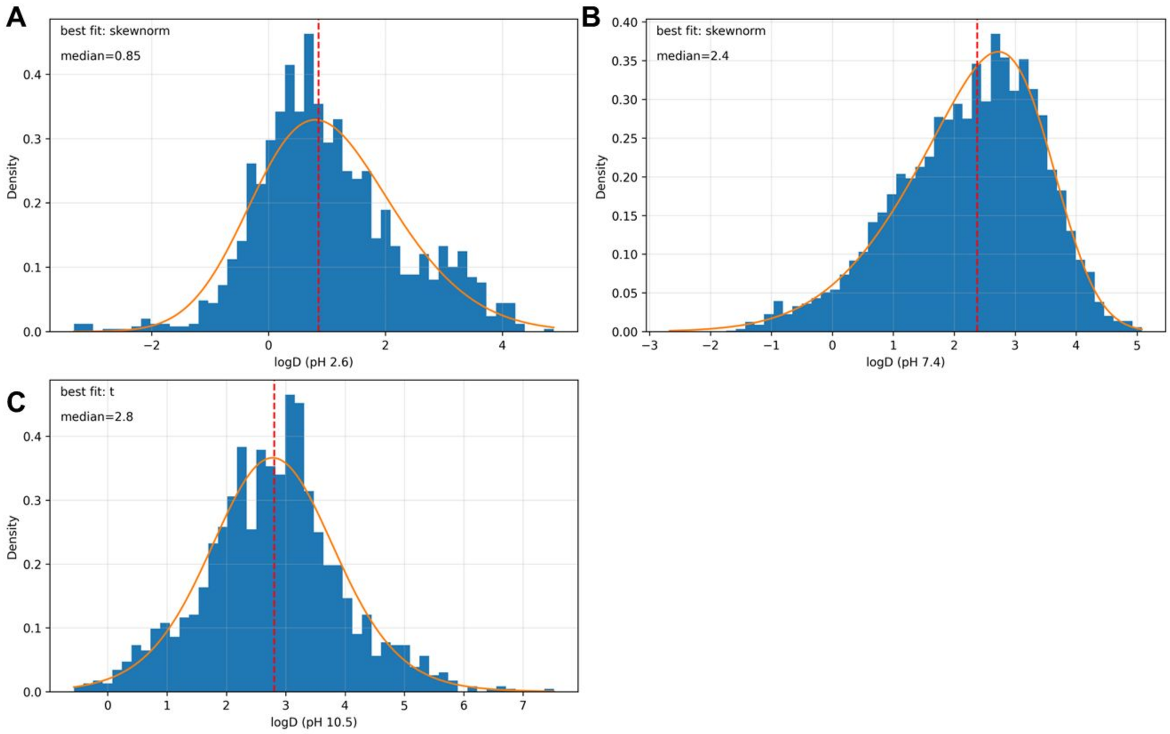

Figure S2. Distribution of logD values at pH 2.6 (A), 7.4 (B), 10.5 (C) with fitted probability density and median.

Aqueous solubility values (logS) span more than ten orders of magnitude, with the majority of compounds clustered in the poorly soluble regime (Figure S3). The distribution is strongly skewed toward lower solubility values, with a median −2.7, consistent with typical medicinal chemistry datasets. This pronounced skewness underscores the intrinsic difficulty of solubility prediction and highlights the importance of modeling approaches that remain stable across wide dynamic ranges.

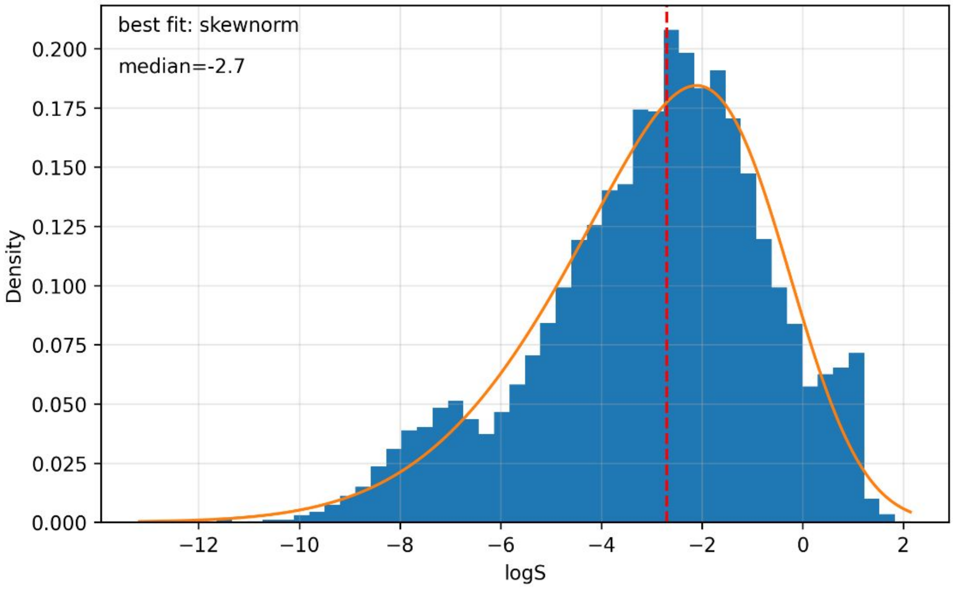

Figure S3. Distribution of logS values with fitted probability density and median.

Acid–base properties were analyzed using two separate macroscopic descriptors: the most acidic pKa and the most basic pKa for each molecule. Both distributions exhibit broad ranges and multimodal characteristics. The most acidic pKa values cluster primarily in the weakly acidic to neutral range, with a median around 6.3 (Figure S4A), while the most basic pKa values extend into strongly basic regimes, with a median 7.0 (Figure S4B). The overlapping yet asymmetric distributions reflect the presence of multiple ionizable sites and diverse functional groups across the dataset. Importantly, these macroscopic labels collapse multiple microscopic protonation states and tautomeric forms into single observable values, contributing to distributional complexity and intrinsic noise.

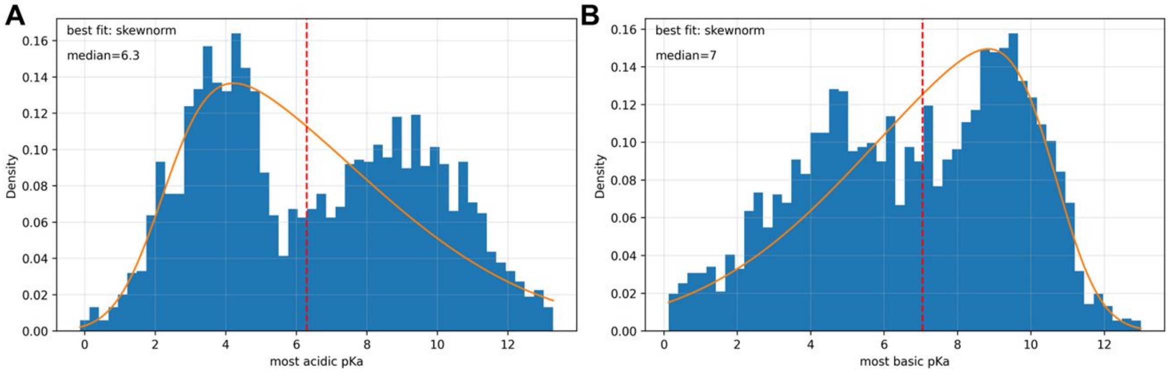

Figure S4. Distribution of most acidic (A) and basic (B) pKa values with fitted probability density and median.

Overall, the observed endpoint distributions are representative of realistic drug-like chemical space and are characterized by wide dynamic ranges, skewness, and non-Gaussian behavior. The observed skewness and heavy-tailed endpoint distributions motivated the use of cross-validated evaluation and complementary performance metrics, combining error-based measures in physicochemical units (RMSE) with variance-explained statistics (Q2, R2), and emphasizing cautious interpretation for acid-base equilibria where label ambiguity is inherent to the experimental definitions.

2.5 Definition of the applicability domain

2.5.1 Overall dataset composition

Prior to model development, the chemical space of each dataset was systematically characterized to assess dataset diversity and to define the applicability domain of subsequent predictive models. The analysis focused on basic structural and physicochemical descriptors capturing molecular size, polarity, and conformational flexibility.

For each endpoint-specific dataset, molecular weight (MW), topological polar surface area (TPSA), heavy atom count, and number of rotatable bonds were analyzed. These descriptors were selected as they provide complementary, low-dimensional summaries of chemical space that are largely orthogonal to the modeled endpoints.

2.5.2 Distribution of physicochemical descriptors

For each dataset, univariate distributions of MW, TPSA, heavy atom count, and number of rotatable bonds were examined using histogram-based summaries. Median values, interquartile ranges (IQR), and 5th-95th percentile ranges were used to describe central tendency and dispersion, providing a robust characterization insensitive to extreme outliers.

The logP (Figure S5) and logS (Figure S6) datasets span a broad chemical space, encompassing small to medium-sized organic molecules. The logP dataset exhibits a wider molecular weight and hydrophobicity range, consistent with its focus on neutral partitioning behavior. In contrast, the logS dataset is shifted toward lower molecular weights and higher polarity, reflecting the intrinsic solubility bias toward more polar and less conformationally flexible compounds.

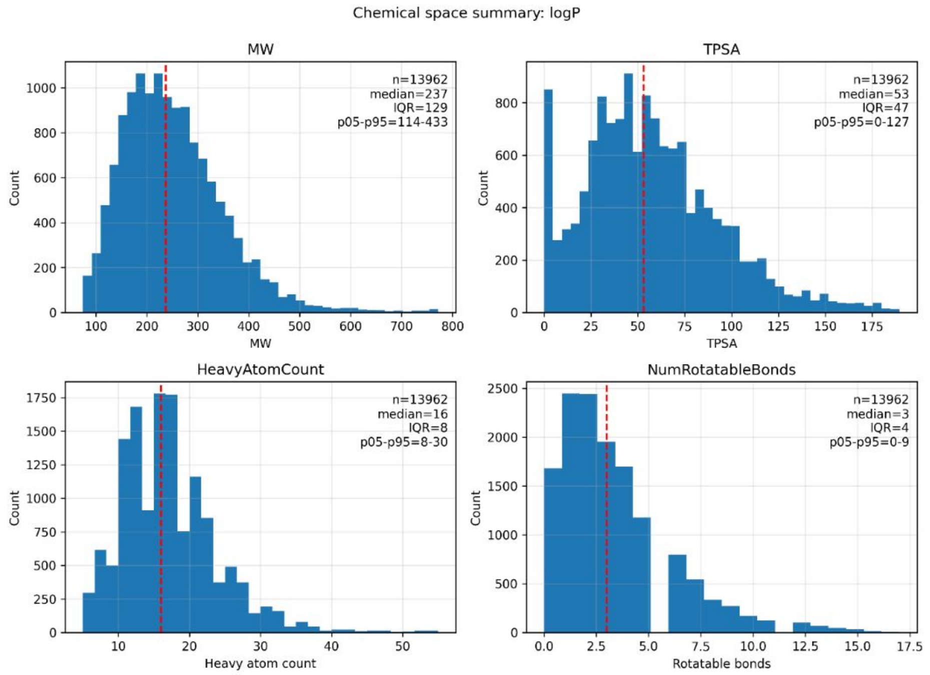

Figure S5. Chemical space characterization of the logP dataset. Distributions of molecular weight (MW), topological polar surface area (TPSA), heavy atom count, and number of rotatable bonds are shown. Dashed red lines indicate median values. Dataset size and summary statistics are reported in each panel.

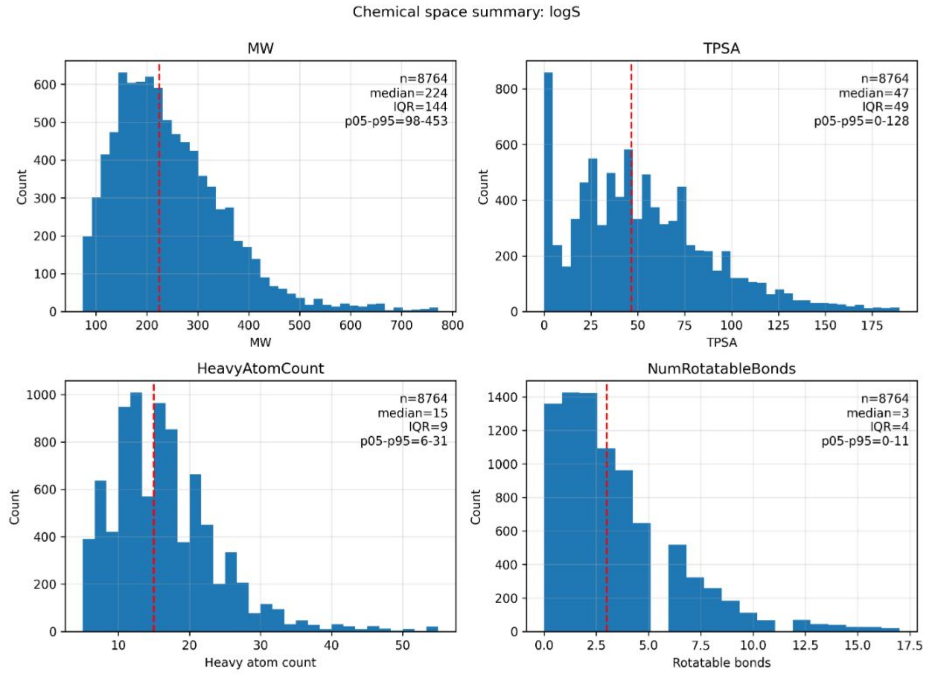

Figure S6. Chemical space characterization of the logS dataset. Distributions of molecular weight (MW), topological polar surface area (TPSA), heavy atom count, and number of

rotatable bonds are shown. Dashed red lines indicate median values. Dataset size and summary statistics are reported in each panel.

The logD datasets at pH 2.6 (Figure S7), 7.4 (Figure S8), and 10.5 (Figure S9) show closely related but non-identical chemical spaces. While median molecular weights and heavy atom counts remain comparable across pH conditions, shifts in TPSA and rotatable bond distributions reflect changes in ionization-dependent partitioning behavior. The physiological pH 7.4 dataset covers the broadest chemical space, whereas the acidic and basic conditions are moderately narrower.

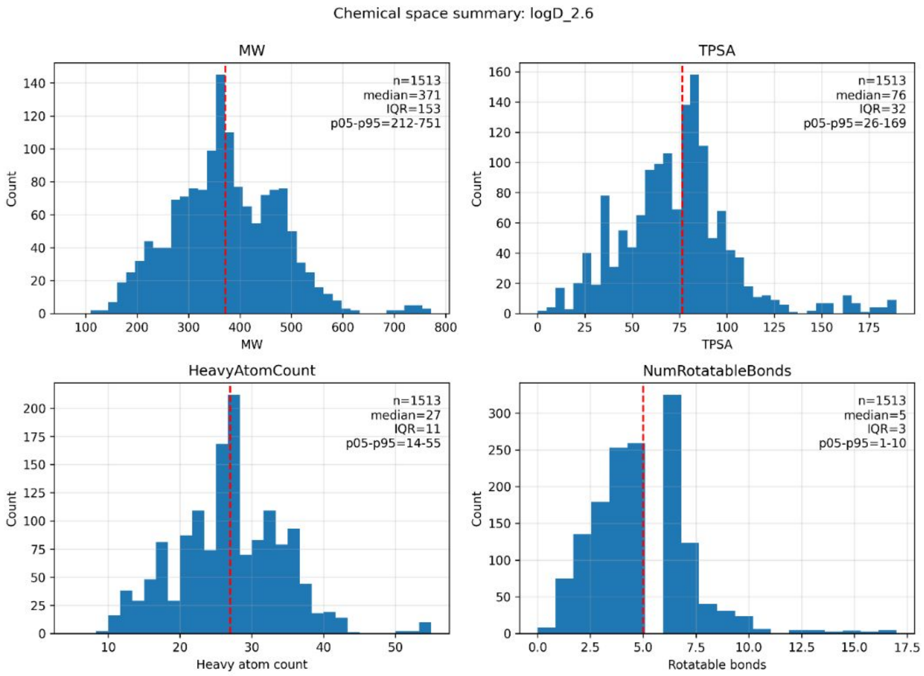

Figure S7. Chemical space characterization of the logD (pH 2.6) dataset. Distributions of molecular weight (MW), topological polar surface area (TPSA), heavy atom count, and number of rotatable bonds are shown. Dashed red lines indicate median values. Dataset size and summary statistics are reported in each panel.

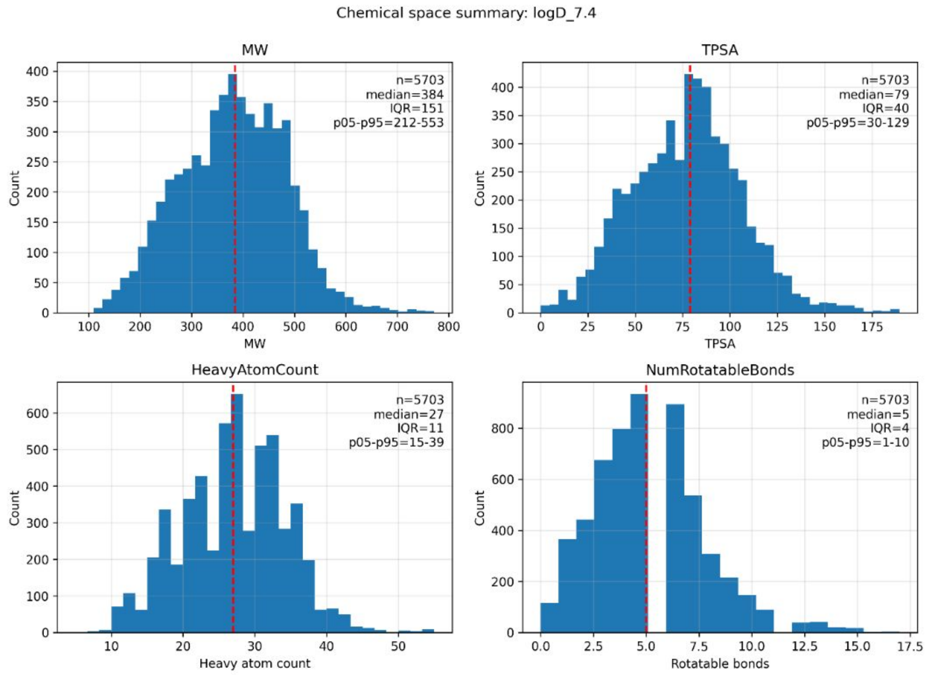

Figure S8. Chemical space characterization of the logD (pH 7.4) dataset. Distributions of molecular weight (MW), topological polar surface area (TPSA), heavy atom count, and number of rotatable bonds are shown. Dashed red lines indicate median values. Dataset size and summary statistics are reported in each panel.

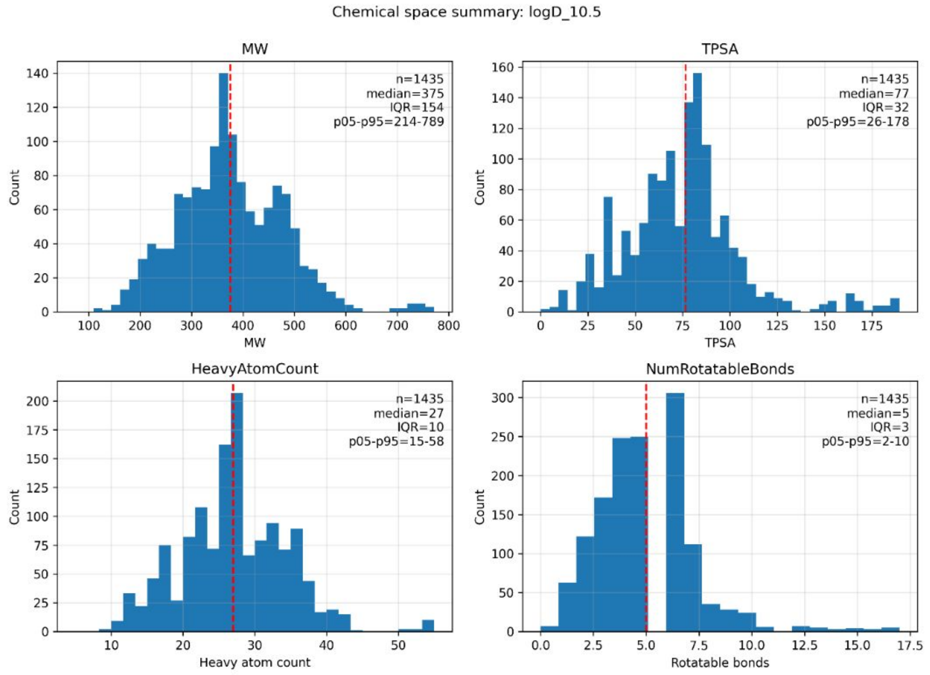

Figure S9. Chemical space characterization of the logD (pH 10.5) dataset. Distributions of molecular weight (MW), topological polar surface area (TPSA), heavy atom count, and number

of rotatable bonds are shown. Dashed red lines indicate median values. Dataset size and summary statistics are reported in each panel.

The most acidic (Figure S10) and most basic (Figure S11) pKa datasets represent two distinct but related chemical spaces. Compounds contributing to the most acidic pKa endpoint are generally smaller and less flexible, whereas the most basic pKa dataset includes a broader range of molecular sizes and polar surface areas. These differences reflect the underlying structural requirements for acidic versus basic ionizable functionalities.

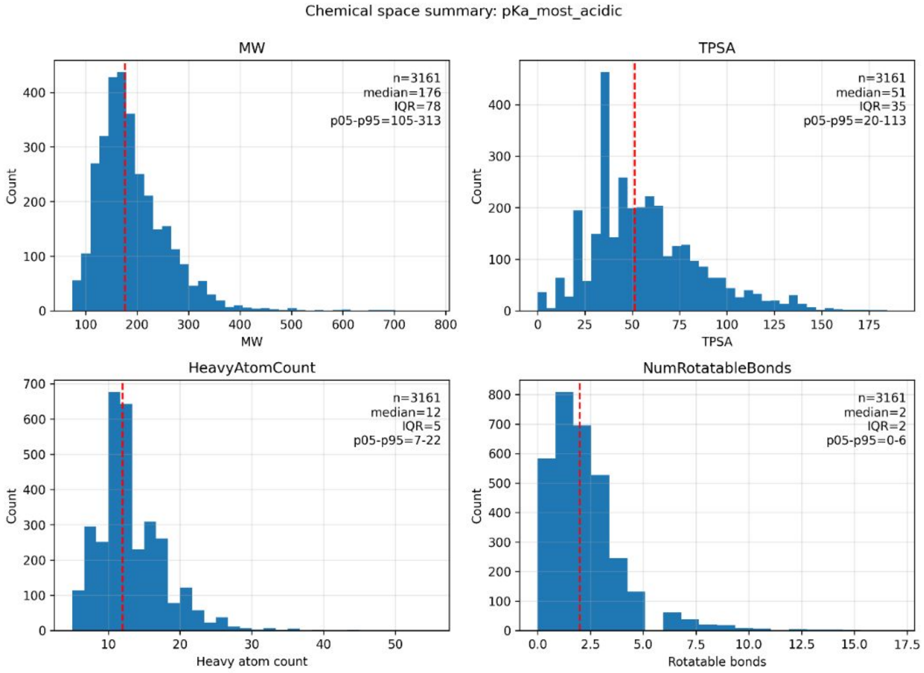

Figure S10. Chemical space characterization of the most acidic pKa dataset. Distributions of molecular weight (MW), topological polar surface area (TPSA), heavy atom count, and number of rotatable bonds are shown. Dashed red lines indicate median values. Dataset size and summary statistics are reported in each panel.

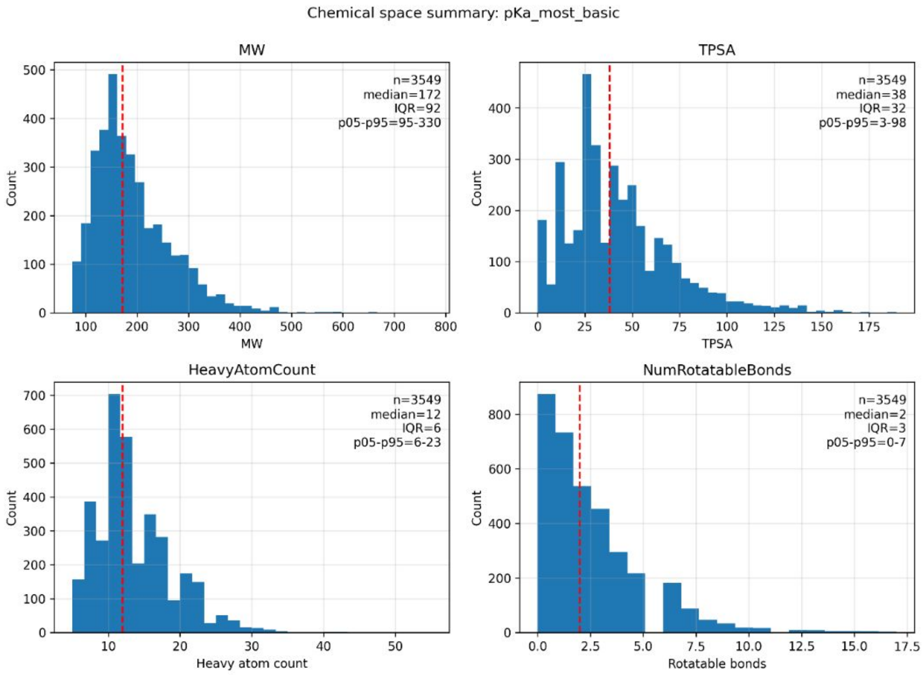

Figure S11. Chemical space characterization of the most basic pKa dataset. Distributions of molecular weight (MW), topological polar surface area (TPSA), heavy atom count, and number of rotatable bonds are shown. Dashed red lines indicate median values. Dataset size and summary statistics are reported in each panel.

2.5.3 Definition of the applicability domain

Based on the observed distributions of MW, TPSA, heavy atom count, and number of rotatable bonds, the applicability domain of each dataset was defined empirically. Compounds falling within the central percentile ranges of these descriptors are considered to lie within the chemical space adequately represented by the training data.

Predictions for molecules substantially outside these descriptor ranges, particularly at the extremes of molecular size, polarity, or flexibility, should be interpreted with caution, as such compounds are underrepresented or absent in the underlying datasets.

3 Molecular Representations

3.1 ECFP4 fingerprint generation

Extended-connectivity fingerprints of the ECFP4 type were generated using the RDKit cheminformatics toolkit. The fingerprints were computed as hashed binary vectors with a fixed length of 2048 bits, employing the Morgan algorithm with a radius of 2, corresponding to the ECFP4 formalism. Chirality information was explicitly included during fingerprint generation

to ensure that stereochemical features contributed to the molecular representation. For each molecule, the resulting fingerprint was encoded as a bit vector, subsequently converted into a tabular representation suitable for downstream machine-learning workflows, with each bit position treated as an independent binary feature.

3.2 NMR vectors generation

For the prediction of ^{1}H and ^{13}C NMR spectra, a Java-based standalone predictor built upon the NMRshiftDB2 database was employed. The tool utilizes HOSE-code pattern matching to assign averaged chemical shift values to atoms, based on a precompiled .csv file containing the complete set of HOSE codes with their corresponding chemical shifts. The predictor internally invokes the org.openscience.nmrshiftdb Java class and is executed locally via Python–Java system integration. The output consists of lists of predicted chemical shift values associated with individual hydrogen or carbon atoms in the molecule. As these outputs do not represent conventional continuous NMR spectra but rather ordered sets of discrete chemical shift values, a dedicated bucketing strategy was implemented to convert them into fixed-length numerical vectors suitable for machine-learning applications. A custom module was developed to partition the relevant chemical shift ranges into uniformly spaced bins. In this study, the ^{1}H NMR region (–1 to 16 ppm) and the ^{13}C NMR region (–10 to 230 ppm) were each divided into 200 equal-width buckets, following a previously established approach. Initially, all buckets were set to zero, and the predicted chemical shift values were iteratively assigned to their corresponding bins. The intensity of a given bin was incremented by one for each atom contributing to that chemical shift range. For instance, three equivalent protons from a methyl group contributed a value of 3 to the appropriate bucket, whereas two protons from a methylene group resulted in a value of 2. This procedure ensured that all molecular spectra were represented as vectors of identical dimensionality, independent of the number or type of atoms present in the molecule.

3.3 Construction of SpectraPRINTS descriptors

SpectraPRINTS were constructed by the sequential head-to-tail concatenation of three feature vectors associated with a given chemical structure. Specifically, the vector derived from the ^{1}H NMR spectrum was placed first, followed by the vector originating from the ^{13}C NMR spectrum, and finally the vector corresponding to the ECFP4 molecular fingerprint was appended.

3.4 SpectraPRINTS dimensionality and normalization

The individual component vectors consisted of 200, 200, and 2048 elements, respectively, resulting in a final composite representation comprising 2448 features. The resulting data structure was represented as a one-dimensional tensor with a shape of (1, 2448), reflecting a single sample described by 2448 features. This representation did not require additional normalization. The ECFP4 fingerprints are inherently normalized by construction, as they consist of binary-valued features. In contrast, the vectors derived from the NMR spectra are numerical and based on integer values, which predominantly fall within the range of 0 to 12. Applying normalization would therefore convert these discrete integer features into floating-point values, potentially introducing unnecessary numerical noise due to finite-precision rounding, without offering a clear methodological advantage.

3.5 Concatenated spectral–structural representations

The resulting representation encodes two distinct types of information. The first component comprises spectral data, represented as ^{1}H and ^{13}C NMR spectra encoded within a 400-element segment of a numerical vector composed of integer values. The second component consists of hashed structural information in the form of an ECFP4 molecular fingerprint. Consequently, the final tensor, with a shape of (1, 2448), simultaneously captures both spectral and structural features, providing an integrated and well-balanced input representation for machine-learning models.

4 Machine Learning Models and Training Protocol

4.1 Model architectures

All predictive models were based on one-dimensional convolutional neural networks designed for regression tasks, operating directly on the SpectraPRINTS representation. The network architecture followed a modular and fully flexible design, allowing both depth and width of the model to be adjusted automatically. The convolutional part consisted of a variable number of one-dimensional convolutional layers, responsible for learning local patterns and correlations within the concatenated spectral–structural feature vector. These layers were followed by a stack of fully connected layers, which performed nonlinear feature integration and regression to a single continuous output. Activation functions, regularization schemes, dropout rates, batch normalization usage, kernel sizes, strides, and weight initialization strategies were treated as tunable components of the architecture rather than fixed design choices. As a result, the final

network topology was not predefined but instead discovered through automated optimization, enabling the model to adapt its complexity to the underlying data structure.

4.2 Training procedure and hyperparameters

Model training was performed using supervised learning with a mean squared error objective function. The optimization process relied on modern gradient-based optimizers and supported mixed-precision arithmetic when GPU acceleration was available. Crucially, hyperparameter optimization was conducted using the Optuna framework, which was employed not only to tune classical training parameters such as learning rate, batch size, optimizer type, or regularization strength, but also to optimize the entire network architecture jointly with the training protocol. This included the number of convolutional and fully connected layers, filter counts, kernel sizes, dropout rates, activation functions, and learning-rate scheduling strategies. Early stopping based on validation loss was systematically applied to prevent overfitting, and learning-rate schedulers were optionally activated to stabilize convergence. Poorly performing configurations were efficiently discarded using Optuna's pruning mechanism, ensuring computational resources were focused on the most promising regions of the hyperparameter space.

4.3 Cross-validation and data splitting strategy

To ensure a robust, unbiased, and fully reproducible evaluation of model performance, a hierarchical and deterministic data-splitting strategy was employed. For each endpoint, the compared molecular representations were constructed for the same curated set of compounds and therefore shared an identical target column, sample count, and sample ordering within that endpoint-specific dataset. Consequently, train/test indices were generated once per endpoint using a fixed random seed and then reused unchanged across all compared molecular representations, ensuring that performance differences reflected descriptor choice rather than differences in sample composition.

At the highest level, the complete dataset was partitioned into a training set comprising 90% of the samples and an independent held-out test set comprising the remaining 10%. This split was generated once using a fixed random seed, and the resulting sample indices were explicitly stored as external index files. These indices were subsequently reused unchanged across all compared descriptor sets, optimization runs, and model architectures within each endpoint. As a result, the held-out test set was identical across all experiments, ensuring that performance

comparisons between different feature representations and modeling approaches were conducted on exactly the same test samples.

The held-out test set was strictly isolated and never accessed during hyperparameter optimization or model selection. All optimization procedures, including hyperparameter tuning and architectural exploration, were performed exclusively on the training portion of the data. Within this training set, k-fold cross-validation was employed during optimization to estimate generalization performance and guide the selection of optimal model configurations.

For final model training, the training set was further subdivided into sub-training and validation subsets using a fixed random seed. This internal validation split was used solely to control early stopping criteria and learning-rate scheduling. After optimization, the final model was retrained on the full training set and evaluated once on the untouched held-out test set. This multi-level and fully deterministic splitting strategy ensured a strict separation between training, validation, and testing phases, minimized the risk of information leakage, and guaranteed full reproducibility and comparability of all reported results.

5 Model Evaluation Metrics

Model performance was assessed using a set of complementary regression metrics selected to quantify both absolute prediction error and the proportion of variance explained by the models. All metrics were computed in a consistent and reproducible manner within the training and validation pipeline, ensuring comparability across different model architectures and optimization trials.

5.1 Root-mean-square error (RMSE)

The primary optimization and evaluation metric used throughout this study was the root-mean-square error (RMSE). RMSE is defined as the square root of the mean of the squared differences between predicted and observed values and provides a direct measure of the average prediction error expressed in the same units as the target variable. Owing to its quadratic penalization of larger errors, RMSE is particularly sensitive to outliers and therefore well suited for detecting systematic prediction failures. Within the present pipeline, RMSE served as the objective function during hyperparameter optimization and was computed on validation folds during cross-validation. Mean RMSE values across folds were used to rank candidate models, while final RMSE values were reported for both cross-validation and the independent test set, providing a transparent measure of predictive accuracy.

5.2 Cross-validated coefficient of determination (Q^2)

The predictive robustness of the models was further quantified using the cross-validated coefficient of determination, Q^2. This metric is defined analogously to the classical coefficient of determination but is calculated on predictions obtained from cross-validation, thereby reflecting true out-of-sample performance. Q^2 measures the fraction of variance in the observed data that is explained by the model when applied to unseen samples, with values closer to unity indicating stronger predictive power. In the implemented workflow, Q^2 was computed for each fold of the cross-validation procedure and subsequently averaged to yield a global cross-validated estimate. This approach provided an additional safeguard against overfitting and enabled a more reliable comparison of models with differing complexity.

5.3 Coefficient of determination (R^2)

The coefficient of determination (R^2) was employed as a complementary descriptive metric to characterize goodness of fit. R^2 quantifies the proportion of variance in the observed target values that is explained by the model predictions relative to a baseline mean predictor. In the present pipeline, R^2 was primarily reported for the final trained model, both on the training data and on the independent test set. While R^2 was not used directly as an optimization criterion, it served as an intuitive indicator of model explanatory power and facilitated comparison with previously reported models in the literature. The combined use of RMSE, Q^2, and R^2 ensured a balanced and comprehensive evaluation of model performance, capturing accuracy, robustness, and explanatory capacity within a unified framework.

6 Extended Benchmarking Results

6.1 Full performance table for all endpoints

Table S1. Comparative performance of machine-learning models trained on different molecular representations for the prediction of physicochemical properties.

Property	Metric	ECFP4	1H|13C	1H|13C|ECFP4	% of enhacement
logP	RMSE 10CV	0.72	0.69	0.52	27.78
	RMSE TEST	0.68	0.66	0.47	30.88

	**Q2**	0.84	0.86	0.92	9.52
	**R2 TEST**	0.87	0.88	0.94	8.05
**logD pH 2.6**	**RMSE 10CV**	0.62	0.63	0.55	11.61
	**RMSE TEST**	0.76	0.52	0.51	32.89
	**Q2**	0.74	0.74	0.80	8.11
	**R2 TEST**	0.78	0.63	0.80	2.56
**logD pH 7.4**	**RMSE 10CV**	0.75	0.82	0.68	9.33
	**RMSE TEST**	0.74	0.85	0.62	16.22
	**Q2**	0.61	0.54	0.67	9.84
	**R2 TEST**	0.62	0.50	0.67	8.06
**logD pH 10.5**	**RMSE 10CV**	0.64	0.76	0.64	0.00
	**RMSE TEST**	0.76	0.64	0.46	39.47
	**Q2**	0.68	0.58	0.69	1.47
	**R2 TEST**	0.58	0.63	0.83	42.54
**logS**	**RMSE 10CV**	1.10	1.04	0.92	16.36
	**RMSE TEST**	1.15	1.07	0.93	19.13
	**Q2**	0.78	0.80	0.85	8.97
	**R2 TEST**	0.78	0.81	0.86	10.26
**pKa (acid)**	**RMSE 10CV**	1.64	2.02	1.67	1.83
	**RMSE TEST**	1.68	1.72	1.73	2.98
	**Q2**	0.72	0.58	0.71	-1.39
	**R2 TEST**	0.68	0.69	0.66	-2.94
**pKa (base)**	**RMSE 10CV**	1.45	1.74	1.40	-3.45
	**RMSE TEST**	1.16	1.48	1.18	1.72
	**Q2**	0.74	0.62	0.76	2.70
	**R2 TEST**	0.83	0.72	0.82	-1.20

The table reports the predictive performance of models based on ECFP4 fingerprints, NMR-derived spectral descriptors ($^1H|^{13}C$), and their combined representation ($^1H|^{13}C|ECFP4$) for

lipophilicity (logP), distribution coefficients (logD at pH 2.6, 7.4, and 10.5), aqueous solubility (logS), and acid–base dissociation constants (pKa, most acidic and most basic sites). Model accuracy is evaluated using root-mean-square error obtained from tenfold cross-validation (RMSE 10CV) and from predictions on an independent test set (RMSE TEST). Predictive robustness is quantified by the cross-validated coefficient of determination (Q^2), while generalization performance is assessed using the coefficient of determination calculated on the test set (R^2 TEST). The percentage of enhancement reflects the relative metric performance rise achieved by augmenting the ECFP4 representation with ^{1}H and ^{13}C NMR spectral information, using the ECFP4-only model as the reference. This metric directly evaluates the contribution of spectral descriptors beyond classical structural fingerprints..

6.2 Parity plots and error distributions

Model performance and reliability were visualized using paired diagnostic panels comprising a parity plot and an embedding-based applicability domain (eAD) analysis. For each endpoint and model variant, these panels provide an interpretable summary of predictive accuracy on the independent test set together with a complementary assessment of whether predictions are produced within the model's learned domain of applicability.

In the left-hand panel, predictive accuracy on the held-out test set is shown as a parity (scatter) plot, where experimental values are plotted against the corresponding model predictions. Agreement between prediction and experiment is indicated by clustering of points along the identity line, whereas systematic deviations reveal bias, compression or expansion of the predicted range, and local regions of poor performance. In addition to overall dispersion, these plots allow rapid identification of heteroscedastic behavior, i.e., cases where prediction error varies across the response range, which is not readily captured by aggregate metrics alone.

The right-hand panel presents an embedding-based applicability domain plot derived from the internal latent representation learned by the neural network. Each point corresponds to one training sample. The x-axis reports the Mahalanobis distance computed in the latent space of the final hidden layer, i.e., in the feature embedding that directly drives the regression output, while the y-axis shows the standardized residual defined as (y_true − y_pred)/RMSE. A vertical threshold in Mahalanobis distance delineates samples considered outside the learned domain in the latent representation, whereas horizontal reference lines at ±3 standardized residual units mark samples exhibiting unusually large prediction errors relative to the model's typical performance. Samples located within the central horizontal band and to the left of the

Mahalanobis threshold correspond to cases that are both well described by the model and lie within its applicability domain, whereas points beyond the distance threshold are treated as out-of-domain, and large absolute standardized residuals indicate poor agreement between prediction and experiment.

This latent-space eAD analysis provides a practical alternative to the classical Williams plot based on leverage in the original descriptor space, which can become ill-defined or non-informative for deep neural networks and high-dimensional inputs, particularly when the feature-to-sample ratio is unfavorable. By operating in the representation actually used by the network, the Mahalanobis eAD plot reflects the geometry of the data in the learned embedding and therefore yields a more meaningful assessment of prediction reliability for modern deep-learning architectures trained on composite inputs such as NMR-derived vectors and structural fingerprints.

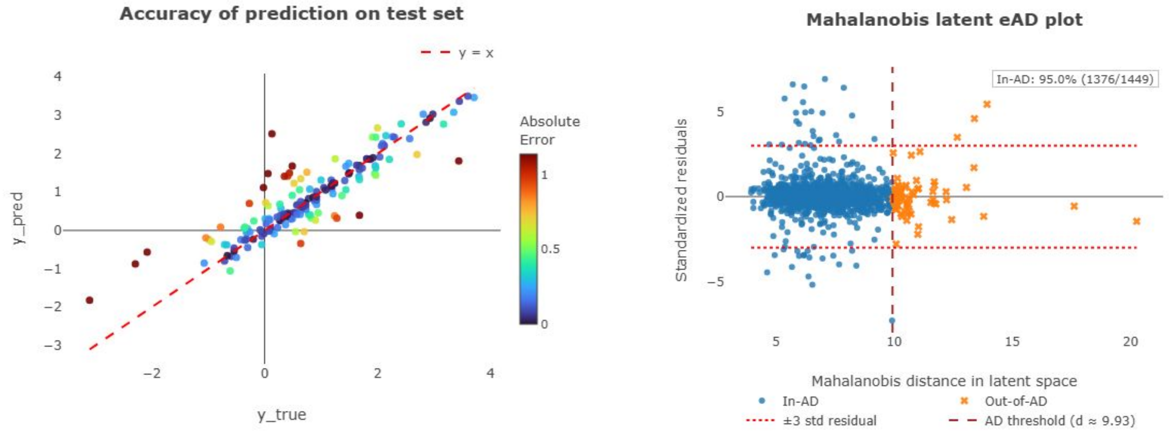

Figure S12. Parity plot and embedding-based applicability domain (eAD) analysis for the prediction of logD at pH 2.6.

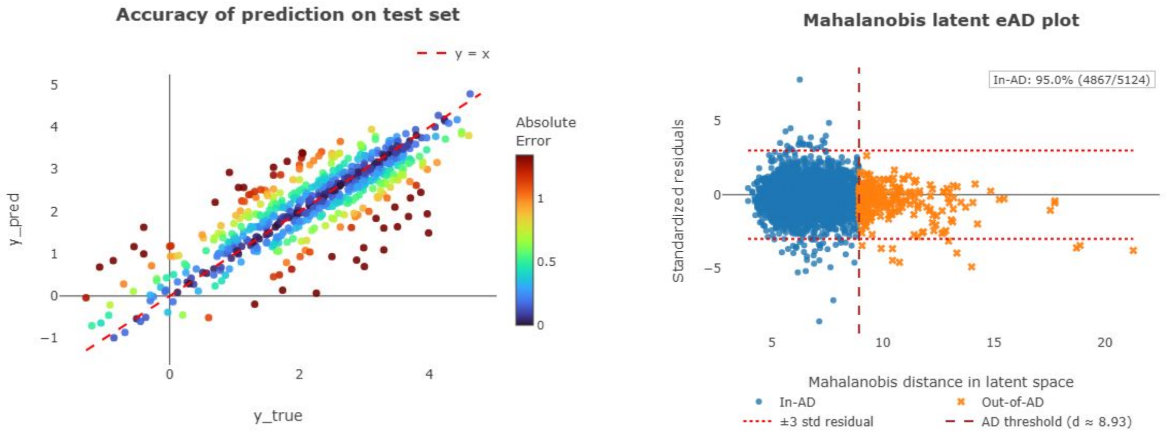

Figure S13. Parity plot and embedding-based applicability domain (eAD) analysis for the prediction of logD at pH 7.4.

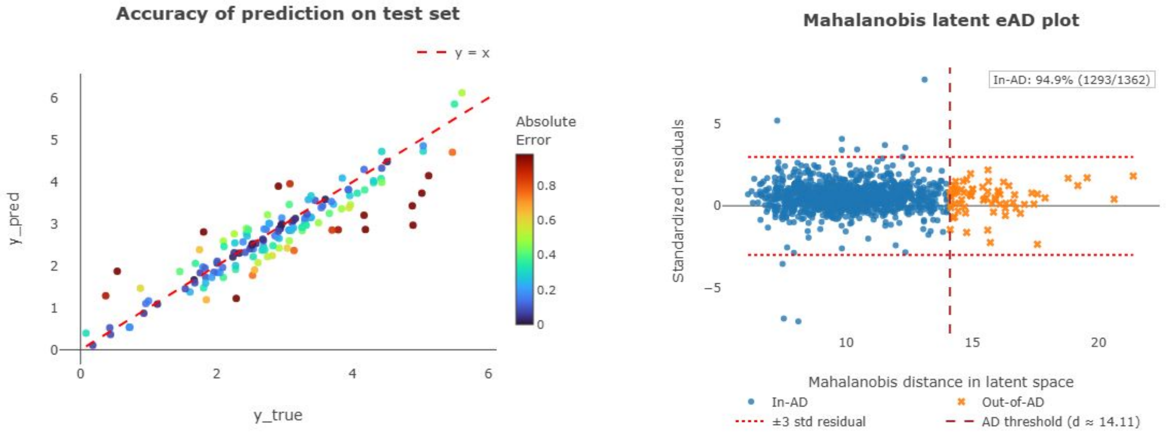

Figure S14. Parity plot and embedding-based applicability domain (eAD) analysis for the prediction of logD at pH 10.5.

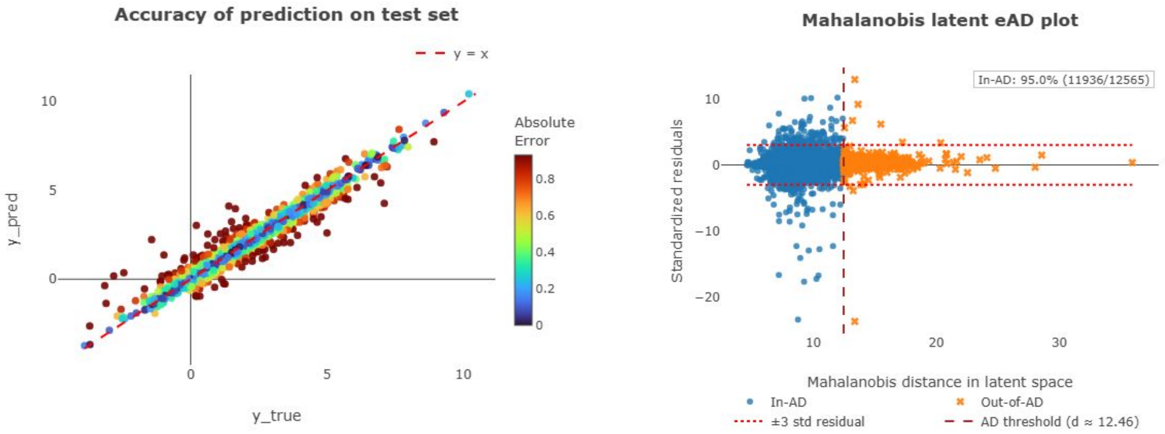

Figure S15. Parity plot and embedding-based applicability domain (eAD) analysis for the prediction of logP.

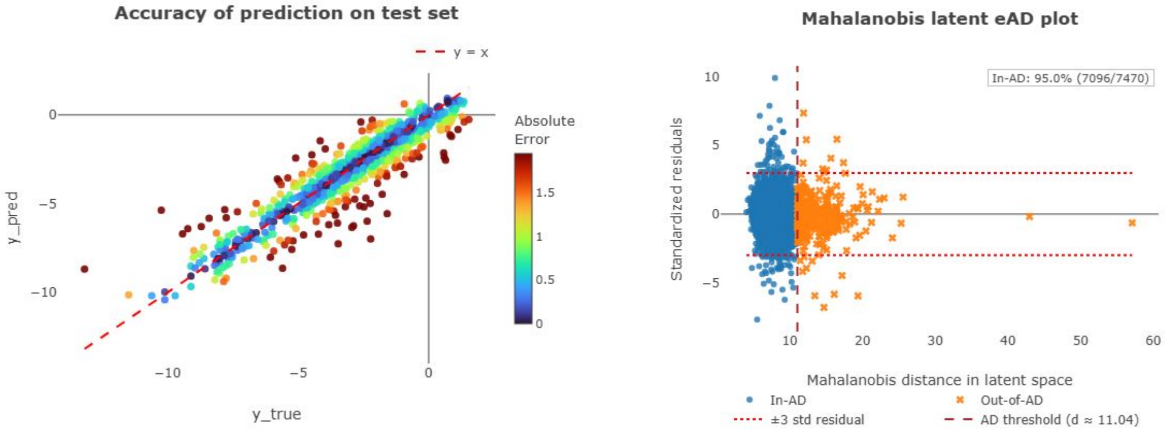

Figure S16. Parity plot and embedding-based applicability domain (eAD) analysis for the prediction of logS.

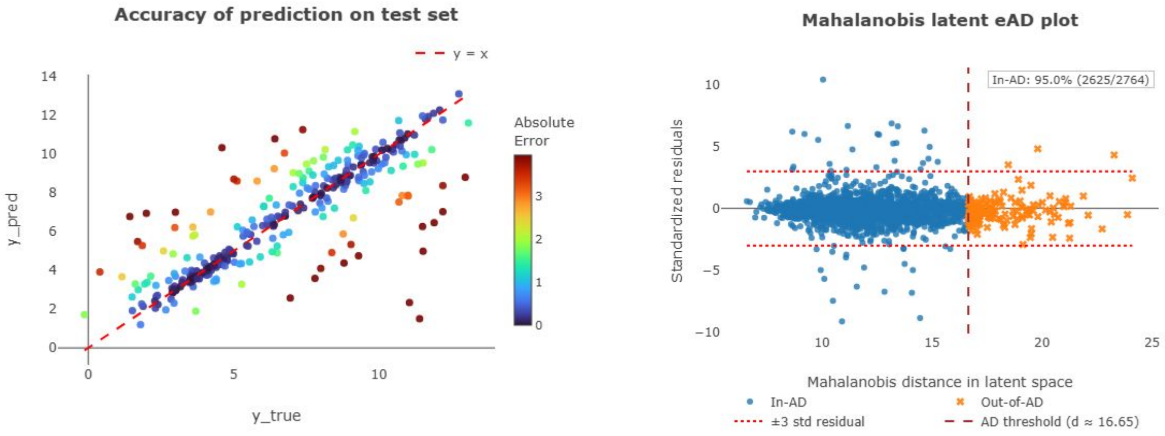

Figure S17. Parity plot and embedding-based applicability domain (eAD) analysis for the prediction of pKa (most acidic).

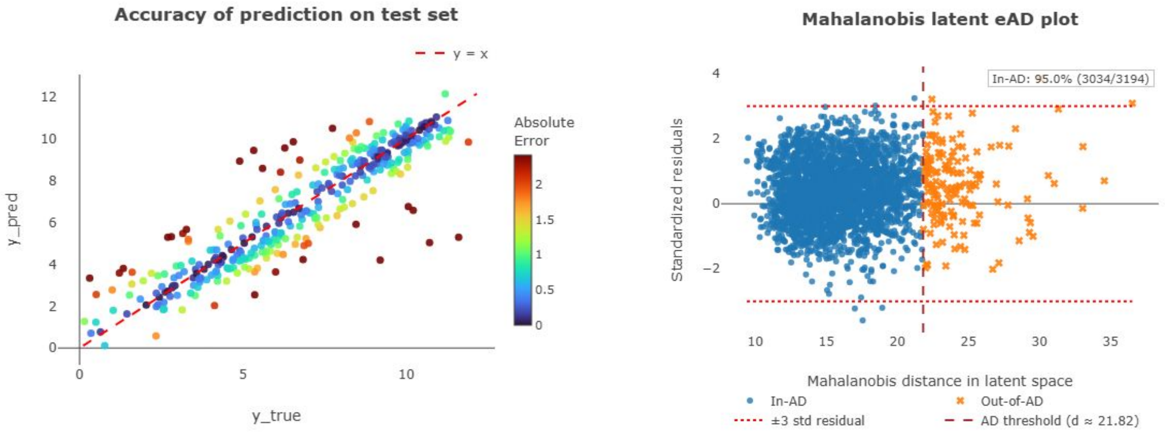

Figure S18. Parity plot and embedding-based applicability domain (eAD) analysis for the prediction of pKa (most basic).

6.3 Endpoint-specific performance analysis

A detailed endpoint-specific analysis reveals that the impact of integrating NMR-derived spectral information with structural fingerprints strongly depends on the physicochemical nature of the predicted property. For lipophilicity (logP), the combined $^{1}H|^{13}C|ECFP4$ representation leads to a pronounced and consistent reduction in prediction error, observed both in cross-validation and on the independent test set. The simultaneous improvement in RMSE, Q^2, and R^2 TEST indicates that spectral descriptors capture complementary information related

to electronic distribution and local chemical environments that is not fully encoded by topological fingerprints alone.

A similar, albeit more nuanced, trend is observed for logD predictions. At all investigated pH values, the inclusion of NMR data improves test-set RMSE, with particularly strong gains at pH 10.5, where a substantial increase in R^2 TEST accompanies a marked reduction in RMSE TEST. This behavior suggests that spectral information is especially informative for properties influenced by ionization equilibria and protonation states, which affect local electronic environments and are indirectly reflected in NMR chemical shifts. The discrepancy between cross-validated and test-set improvements for certain pH values further highlights the sensitivity of logD predictions to data distribution and emphasizes the importance of independent test evaluation.

For aqueous solubility (logS), the combined representation yields systematic improvements across all reported metrics. The concurrent decrease in RMSE and increase in both Q^2 and R^2 TEST point to a robust enhancement of predictive performance. Given the strong dependence of solubility on intermolecular interactions, polarity, and functional group accessibility, these results indicate that NMR-derived descriptors effectively complement structural fingerprints by encoding aspects of molecular behavior that extend beyond purely topological features.

In contrast, the benefits of spectral information are less pronounced for pKa prediction. For both the most acidic and most basic dissociation constants, the combined representation does not consistently outperform ECFP4-based models, and in some cases leads to marginally inferior Q^2 or R^2 TEST values. This observation suggests that pKa is predominantly governed by well-defined local structural motifs and ionizable functional groups, which are already efficiently captured by classical fingerprint representations. The limited or negative enhancement observed here indicates that, for this endpoint, NMR spectral descriptors may provide little additional non-redundant information within the current modeling framework.

Overall, this endpoint-specific analysis demonstrates that ^1H and ^{13}C NMR data are not universally beneficial but act as a selectively informative modality. Their integration with structural fingerprints leads to substantial performance gains for properties governed by global electronic effects and intermolecular interactions, while offering limited advantage for endpoints dominated by localized structural determinants. This behavior supports the

interpretation that spectral descriptors encode complementary physicochemical information rather than serving as a redundant alternative to established fingerprint-based representations.

7 Acid–Base Modeling and Handling of Most Acidic and Most Basic pKa endpoints

7.1 Acid–base site classification

Acid–base behavior is treated within the NMR-AI platform as a classification and selection problem rather than a full microstate-resolved protonation modeling task. Molecular inputs are first analyzed to identify the presence of ionizable acidic and/or basic sites, enabling assignment of each compound to one of four categories: acidic, basic, amphoteric, or neutral. This classification step determines how model-predicted acid–base values are interpreted and reported downstream.

Ionizable site classification is performed using a two-stage strategy that combines complementary sources of information. First, rule-based substructure recognition is applied using predefined SMARTS patterns to detect common acidic and basic functional groups. In parallel, microstate enumeration is carried out using Dimorphite-DL within a restricted pH window (default pH 7.4 ± 0.6) to identify whether positively and/or negatively charged microstates are accessible under near-physiological conditions. The final acid–base label is obtained by combining the outputs of both approaches using a deterministic decision rule, ensuring robust classification across diverse chemical scaffolds.

7.2 Selection of most acidic and most basic pKa values

Machine-learning models within the platform independently predict two macroscopic pKa-related quantities per molecule: (i) the most acidic pKa, corresponding to the lowest pKa among predicted acidic ionization events, and (ii) the most basic pKa, corresponding to the highest pKa of the conjugate acid associated with basic ionization sites. These values are predicted directly by trained regression models and do not rely on enumeration of individual microstates or tautomer populations.

Based on the acid–base classification described above, a single macroscopic acid–base value (pKa_final) is selected for reporting when required by downstream analyses or decision metrics. For compounds classified as acidic, the most acidic pKa is reported; for compounds classified

as basic, the most basic pKa is reported. For amphoteric molecules, selection follows a predefined priority rule consistent with the intended physicochemical context, while neutral molecules are assigned no pKa_final value. This reduction yields a single interpretable descriptor suitable for integration with other predicted properties, such as logD or CNS-MPO.

7.3 Limitations of macroscopic pKa labeling

The acid–base handling strategy implemented here is intentionally simplified and operates at the level of macroscopic pKa extremes. As such, it collapses potentially complex protonation behavior involving multiple microstates, tautomers, and site-specific equilibria into a single representative value. This simplification does not capture pH-dependent population distributions, coupling between ionization sites, or context-dependent shifts in dominant microstates.

Consequently, while the reported pKa values are suitable for comparative analysis, large-scale benchmarking, and integration into composite metrics such as CNS-MPO, they should not be interpreted as substitutes for detailed microstate-resolved pKa modeling. In particular, endpoints that are highly sensitive to protonation equilibria may require more sophisticated treatment beyond global descriptors and macroscopic labeling. These limitations are reflected in the observed performance trends for pKa-related predictions and motivate future extensions toward microstate-aware representations and modeling strategies.

From a mechanistic perspective, nuclear magnetic resonance provides a fundamentally local probe of acid–base equilibria. During NMR titration experiments, pKa (or pKb) values are inferred by monitoring chemical shift changes of nuclei in the immediate vicinity of the protonation or deprotonation site, thereby directly reporting on the corresponding microscopic acid–base event rather than on a global molecular property.[4] As a result, NMR-derived observables are intrinsically sensitive to individual protonation microstates and their local electronic environments, while macroscopic pKa values reported in datasets represent ensemble-averaged quantities collapsing multiple sites, tautomers, and protonation pathways into a single scalar label. This conceptual mismatch further contributes to the limited performance gains observed for pKa prediction using global molecular representations alone.

8 NMR-AI Platform Description

8.1 Molecular input and representation generation

The NMR-AI platform accepts molecular structures provided as SMILES strings[4] or structure files and enables their transformation into machine-learning-ready numerical representations within a unified workflow. Molecular inputs are processed using established cheminformatics toolkits to generate a range of structural descriptors and fingerprints. Within the platform, molecular representations are organized into three complementary families: (i) structure-based fingerprints derived from molecular graphs, (ii) continuous physicochemical descriptors computed from molecular structure, and (iii) NMR-derived spectral descriptors constructed from predicted chemical shifts. These feature blocks can be generated independently or concatenated in user-defined combinations, and are exported through a unified, consistent tabular schema regardless of the selected representation. Supported representations include circular fingerprints (e.g., ECFP variants),[5] substructure-based fingerprints such as Klekota–Roth,[6] and a broad set of RDKit-derived physicochemical descriptors.[7] All continuous descriptor blocks can be optionally normalized using reusable scaling schemes to ensure consistency across datasets and prediction tasks.

In parallel, NMR-derived SpectraPRINTS descriptors can be generated for the same molecular inputs. All descriptor blocks are constructed deterministically and can be used independently or concatenated into hybrid feature spaces, allowing systematic exploration of representation complementarity without external preprocessing. Users may explicitly select which descriptor blocks are generated and concatenated, enabling systematic evaluation of individual representations and their combinations without modifying code or retraining models externally.

8.2 NMR spectra prediction and SpectraPRINTS construction

For NMR-based representations, the platform uses predicted one-dimensional ^{1}H and ^{13}C chemical shifts. Chemical shifts are predicted using a HOSE-code-based method trained and validated on reference data from the open nmrshiftdb2 resource, enabling scalable generation of shift lists prior to compound synthesis and without requiring physical sample availability.[8–10] In our previous work, predicted shifts were shown to provide equal or superior performance as machine-learning inputs compared with experimentally measured spectra, supporting their use as the default NMR source in the present workflow.[2,11]

Predicted chemical shifts are converted into SpectraPRINTS by fixed, predefined chemical-shift binning followed by histogram aggregation. Specifically, each nucleus is mapped to a fixed ppm window and bin width, and the number of predicted signals falling into each bin is counted to yield a fixed-length integer vector (left-closed, right-open binning). In the current implementation, standard ^{1}H shifts are binned over -1.0 to 17.0 ppm with a bin width of 0.08 ppm, and ^{13}C shifts are binned over -10.0 to 230.0 ppm with a bin width of 1.1 ppm (these thresholds and bin width can be modified). The resulting ^{1}H and ^{13}C NMR vectors can be used independently or concatenated with ECFP4 to form SpectraPRINTS. For batch processing, predicted shifts are produced per molecule and nucleus and stored in structured tabular outputs, which are then deterministically transformed into bucket vectors with standardized column naming (H_* and C_* bins) and a consistent schema suitable for downstream machine-learning pipelines and feature concatenation with structure-based descriptors.

8.3 Property prediction and derived decision metrics

The platform provides prediction of multiple physicochemical properties relevant to molecular design, including lipophilicity (logP), aqueous solubility (logS), pH-dependent distribution coefficients (logD), and acid–base equilibria. Property prediction is performed by querying a fixed set of pretrained machine-learning models developed in this work, operating on predefined molecular representations based on concatenated SpectraPRINTS and ECFP4 fingerprints. No model retraining is performed at runtime, ensuring deterministic and reproducible predictions across batch and interactive workflows.

Acid–base behavior is described using two model outputs corresponding to macroscopic pKa extremes: the most acidic pKa (lowest pKa among ionizable acidic sites) and the most basic pKa (highest pKa of the conjugate acid among ionizable basic sites). To report a single acid–base value per molecule when required (pKa_final), automatic acid–base classification is performed to determine whether the compound behaves predominantly as an acid, a base, both, or neither. This classification employs two complementary backends: (i) RDKit SMARTS-based ionizable site heuristics and (ii) Dimorphite-DL microstate enumeration within a defined pH window (default pH 7.4 +/- 0.6), which identifies the presence of positively and/or negatively charged microstates.[12] The final acid–base label is obtained using a combined decision rule, and a single macroscopic value is selected accordingly (most acidic pKa for acids, most basic pKa for bases). This procedure yields simplified macroscopic estimates that may collapse multiple microstates and tautomers.

In addition to direct property prediction, NMR-AI computes derived decision-support metrics commonly used in medicinal chemistry. PAINS substructure alerts are generated using established substructure filters,[13] and CNS-MPO scores are calculated from predicted physicochemical properties following the original multiparameter optimization framework.[14] Because CNS-MPO depends on predicted acid–base properties, its uncertainty reflects the limitations of macroscopic acid–base assignment and should be interpreted accordingly. Together, primary property predictions and derived metrics support both quantitative assessment and qualitative compound prioritization during early-stage molecular design.

8.4 Interactive live molecular design

Beyond batch prediction, the platform provides an interactive live molecular design environment that supports iterative, user-driven exploration of structural modifications with immediate feedback from pretrained models. Users can edit a starting structure and evaluate successive analogs in a loop, where molecular representations are regenerated on the fly and predictions are updated in real time for all supported physicochemical endpoints (logP, logS, and logD across pH regimes) together with derived decision metrics (PAINS alerts and CNS-MPO). Each iteration is recorded as a structured result entry linking the modified SMILES to the corresponding predicted properties, enabling transparent tracking of structure–property changes across an optimization trajectory.

This interactive workflow facilitates rapid exploration of structure–property relationships by removing the need for repeated external preprocessing, manual feature generation, or separate model execution. By providing consistent outputs across iterations, the live environment enables hypothesis-driven optimization and supports practical compound refinement by allowing users to immediately assess the physicochemical consequences of specific chemical edits.

8.5 Visualization and data export

To support analysis and reporting, NMR-AI provides integrated visualization of molecular structures, predicted properties, and NMR-derived representations. Prior to visualization and downstream prediction, molecular inputs undergo automated structural validation and consistency checks to ensure chemically reasonable representations and to prevent propagation of invalid structures into descriptor generation and model inference. Molecules can be rendered in two-dimensional and three-dimensional formats and predicted $^{1}H|^{13}C$ spectra can be

inspected alongside structural features to facilitate interpretation of SpectraPRINTS-based inputs.

All generated feature blocks (structural fingerprints, continuous descriptors, and SpectraPRINTS), predicted properties, and derived metrics are exportable in standard tabular formats with a consistent column schema, enabling downstream analysis and external machine-learning workflows without additional formatting. For figures, the platform supports direct export of publication-ready graphics using predefined export presets that control key formatting parameters (canvas size, resolution, font scaling, and line weights), providing deterministic outputs suitable for inclusion in manuscripts and presentations.

9 Software Implementation and Reproducibility

9.1 Software libraries and versions

9.1.1 Web application framework

The NMR-AI platform is implemented as a web-based application using the Streamlit framework (version 1.50.0), which provides a lightweight and reproducible interface layer for interactive molecular modeling workflows. Streamlit is used exclusively for user interaction, visualization, and managing of backend computations.

All cheminformatics processing, descriptor generation, and property prediction are executed by deterministic Python (version 3.12) backend modules, and no model retraining or parameter optimization is performed at runtime. The Streamlit-based architecture enables both batch and interactive execution modes while preserving identical prediction logic and outputs across workflows. The application framework therefore, does not affect model behavior or prediction outcomes, but serves solely as an execution and visualization layer.

9.1.2 Cheminformatics and molecular representations

All cheminformatics operations, molecular parsing, and structure-based feature generation were performed using RDKit (version 2024.03.6) and PaDEL-Descriptor (version 0.1.16)[15]. RDKit was used to generate circular fingerprints (ECFP variants), continuous physicochemical descriptors derived from molecular graphs, and to perform SMARTS-based structural validation and acid–base heuristics.

Klekota–Roth substructure fingerprints[6] were generated using the PaDEL-Descriptor software,[15] which provides an established implementation of this fingerprinting scheme based on predefined substructure keys. All fingerprint and descriptor blocks were generated deterministically and applied consistently across all datasets and prediction tasks.

9.1.3 NMR spectra prediction

One-dimensional ^{1}H and ^{13}C NMR chemical shifts were predicted using a HOSE-code-based approach implemented[9] in the nmrshiftdb2[8,10] prediction engine (Java backend, version 17.0.13). The predictor was accessed through a Python interface and calibrated on reference spectra from the nmrshiftdb2 database. Predicted per-atom chemical shifts were subsequently used to construct SpectraPRINTS descriptors through fixed chemical-shift binning, yielding fixed-length numerical representations compatible with downstream machine-learning workflows. No experimentally measured spectra were required, enabling applicability prior to compound synthesis.

9.1.4 Acid–base classification

Automatic acid–base classification was performed using a hybrid strategy combining rule-based substructure recognition and microstate enumeration. SMARTS-based heuristics implemented via RDKit were used to identify common acidic and basic functional groups. In parallel, protonation-state enumeration was carried out using Dimorphite-DL[12] (version 2.0.2) within a restricted pH window centered around physiological conditions. Outputs from both approaches were combined using deterministic decision rules to assign each molecule to acidic, basic, amphoteric, or neutral categories, which guided the selection and reporting of macroscopic pKa values.

9.1.5 Machine learning

All machine-learning models were implemented in Python using scikit-learn (version 1.5.x) and PyTorch (version 2.4.x) as the core computational frameworks. Classical preprocessing steps, including deterministic median imputation of missing values and stratified data splitting, were performed using scikit-learn utilities, ensuring strict separation between training, validation, and test sets without information leakage.

Deep-learning models were implemented using PyTorch and consisted of fully configurable one-dimensional convolutional neural networks (1D CNNs) designed for regression tasks on

fixed-length molecular descriptors. Network architectures were optimized using Optuna (version 3.6.x) with Bayesian hyperparameter optimization and integrated pruning strategies, including median-based and patience-based pruners, to efficiently discard underperforming configurations. Hyperparameters optimized included network depth, convolutional kernel sizes and strides, activation functions, dropout rates, batch normalization, optimizer type (Adam, AdamW, RMSprop, SGD), learning-rate schedules, gradient clipping thresholds, and regularization schemes (L1/L2).

Model training employed k-fold cross-validation with deterministic random seeds and optional mixed-precision training (AMP) on CUDA-enabled devices to ensure numerical stability and reproducibility. Early stopping with rollback to the best validation epoch was applied consistently across all folds and during final model training. Model performance was assessed using RMSE, MAE, Q^2, and Pearson correlation coefficients, with all metrics logged automatically using MLflow (version 2.13.x).

In addition to standard evaluation, the trained neural networks were further analyzed using latent-space diagnostics. Latent representations were extracted from the penultimate network layer and used to construct applicability-domain analyses, including Williams plots and embedding-based Mahalanobis distance metrics, providing a quantitative assessment of model reliability and extrapolation risk. All trained models, hyperparameters, evaluation metrics, diagnostic plots, and prediction outputs were logged as MLflow artifacts, enabling full reproducibility of the reported results. The complete training and optimization workflow is implemented in a standalone Python script available in a public GitHub repository CNN_1D_pytorch.py.

The full source code of the machine-learning pipeline is openly available at:

https://github.com/Prospero1988/NMR-AI_part4

9.2 Reproducibility

All datasets, molecular representations, model configurations, and trained model parameters are versioned and maintained consistently within the NMR-AI platform. Descriptor generation, normalization schemes, and prediction pipelines are deterministic, ensuring that identical inputs yield identical outputs across runs. This design supports independent validation of reported results and facilitates reproducible application of NMR-enhanced molecular modeling workflows.

10 Supplementary References

[1] K. Mansouri, C. M. Grulke, R. S. Judson, A. J. Williams, “OPERA models for predicting physicochemical properties and environmental fate endpoints” *J Cheminform* 2018, *10*, 10.

[2] A. Leniak, W. Pietruś, A. Świderska, R. Kurczab, “From NMR to AI: Do We Need 1H NMR Experimental Spectra to Obtain High-Quality logD Prediction Models?” *J Chem Inf Model* 2025, *65*, 2924–2939.

[3] Z. Wu, B. Ramsundar, E. N. Feinberg, J. Gomes, C. Geniesse, A. S. Pappu, K. Leswing, V. Pande, “MoleculeNet: a benchmark for molecular machine learning” *Chem Sci* 2018, *9*, 513–530.

[4] D. Weininger, “SMILES, a chemical language and information system. 1. Introduction to methodology and encoding rules” *J Chem Inf Comput Sci* 1988, *28*, 31–36.

[5] D. Rogers, M. Hahn, “Extended-Connectivity Fingerprints” *J Chem Inf Model* 2010, *50*, 742–754.

[6] J. Klekota, F. P. Roth, “Chemical substructures that enrich for biological activity” *Bioinformatics* 2008, *24*, 2518–2525.

[7] RDKit: Open-source cheminformatics, 2025.

[8] C. Steinbeck, S. Kuhn, “NMRShiftDB – compound identification and structure elucidation support through a free community-built web database” *Phytochemistry* 2004, *65*, 2711–2717.

[9] S. Kuhn, S. R. Johnson, “Stereo-Aware Extension of HOSE Codes” *ACS Omega* 2019, *4*, 7323–7329.

[10] C. Steinbeck, S. Krause, S. Kuhn, “NMRShiftDB - Constructing a Free Chemical Information System with Open-Source Components” *J Chem Inf Comput Sci* 2003, *43*, 1733–1739.

[11] A. Leniak, W. Pietruś, R. Kurczab, “From NMR to AI: Fusing 1H and 13C Representations for Enhanced QSPR Modeling” *J Chem Inf Model* 2025, *65*, 10323–10337.

[12] P. J. Ropp, J. C. Kaminsky, S. Yablonski, J. D. Durrant, “Dimorphite-DL: an open-source program for enumerating the ionization states of drug-like small molecules” *J Cheminform* 2019, *11*, 14.

[13] J. B. Baell, G. A. Holloway, “New Substructure Filters for Removal of Pan Assay Interference Compounds (PAINS) from Screening Libraries and for Their Exclusion in Bioassays” *J Med Chem* 2010, *53*, 2719–2740.

[14] T. T. Wager, X. Hou, P. R. Verhoest, A. Villalobos, "Central Nervous System Multiparameter Optimization Desirability: Application in Drug Discovery" *ACS Chem Neurosci* 2016, *7*, 767–775.

[15] C. W. Yap, "PaDEL-descriptor: An open source software to calculate molecular descriptors and fingerprints" *J Comput Chem* 2011, *32*, 1466–1474.
